# Functional Insights from the Crystal Structure of the N-Terminal Domain of the Prototypical Toll Receptor

**DOI:** 10.1016/j.str.2012.11.003

**Published:** 2013-01-08

**Authors:** Monique Gangloff, Christopher J. Arnot, Miranda Lewis, Nicholas J. Gay

**Affiliations:** 1 Department of Biochemistry, University of Cambridge, Cambridge, CB2 1GA, UK

## Abstract

*Drosophila melanogaster* Toll is the founding member of an important family of pathogen-recognition receptors in humans, the Toll-like receptor (TLR) family. In contrast, the prototypical receptor is a cytokine-like receptor for Spätzle (Spz) protein and plays a dual role in both development and immunity. Here, we present the crystal structure of the N-terminal domain of the receptor that encompasses the first 201 amino acids at 2.4 Å resolution. To our knowledge, the cysteine-rich cap adopts a novel fold unique to Toll-1 orthologs in insects and that is not critical for ligand binding. However, we observed that an antibody directed against the first ten LRRs blocks Spz signaling in a *Drosophila* cell-based assay. Supplemented by point mutagenesis and deletion analysis, our data suggests that the region up to LRR 14 is involved in Spz binding. Comparison with mammalian TLRs reconciles previous contradictory findings about the mechanism of Toll activation.

## Graphical abstract

**Graphical Abstract:**

**Figure:**
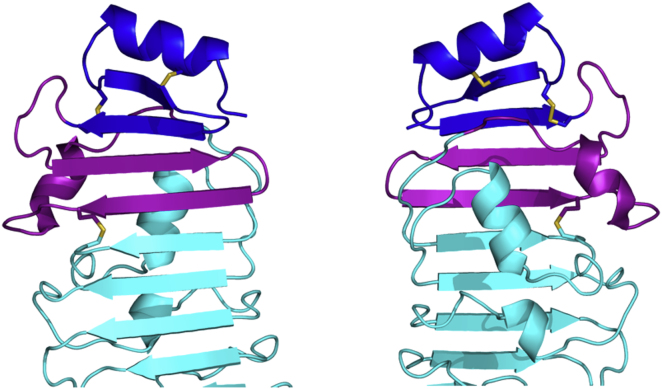

**Highlights:**

► The N-terminal cap of Toll adopts a new fold ► It resembles a duplicated beta hairpin structure with three disulphide bonds ► Toll LRRNT cap is not critical for Spätzle binding ► The first 13 LRRs are sufficient for Spätzle binding

## Teaser

Gangloff et al. describe a crystal structure of the N-terminal domain of the prototypical drosophila Toll receptor at 2.4 Å resolution. The cysteine-rich N-terminal cap adopts a new fold unique to Toll-1 orthologues in insects. Contrary to expectations, it is not critically involved in Späzle binding.

## Introduction

Over the past hundred years, the fruit fly *Drosophila melanogaster* has been an extremely successful model organism that has allowed the characterization of a number of key pathways in both insects and mammals. Genetic screens triggered “stunning” embryos (translated from “Toll” in German) with defects in the dorsoventral axis (Anderson et al., 1985). It was later discovered that the *Toll* gene was critical in the innate immunity of adult flies (Lemaitre et al., 1996). Homologs of Toll have been identified in mammals based on their conserved architecture with leucine-rich repeat (LRR) ectodomains, single-pass transmembrane domains, and intracellular signaling domains (Medzhitov et al., 1997). The latter is shared by Toll and the interleukin-1 receptor (TIR domain) (Gay and Keith, 1991). Mammalian Toll counterparts, the Toll-like receptors (TLRs), have a conserved role in immunity without any involvement in embryonic development. TLRs are true pathogen-recognition receptors that bind directly to a diverse repertoire of microbial signature molecules, ranging from lipopolysaccharide (LPS) for TLR4; lipopeptides for TLRs 2, 1, and 6; nucleic acids for TLRs 3, 7, 8, and 9; and flagella protein for TLR5 (Gay and Gangloff, 2007). In contrast, Toll is a cytokine-like receptor for an endogenous protein Spätzle (Spz) that is unique to insects. Spz is structurally related to mammalian growth factors, such as the nerve growth factor (NGF) (Hoffmann et al., 2008; Mizuguchi et al., 1998). It is expressed as an inactive pro-protein that is targeted by specific protease cascades during development and immunity, respectively. Endoproteolytic processing of Spz triggers a conformational change in its C-terminal active fragment of 106 amino acids (C-106) that engages the receptor and initiates signaling (Arnot et al., 2010). A core set of adaptors, dMyD88, Tube, and Pelle, constitute the immediate postreceptor molecules. Intracellular signaling involves protein assemblies mediated initially by the TIR domains of the receptor and dMyD88 and then via the Death domains (DD) of Tube and Pelle. The latter is a kinase that triggers the phosphorylation of Cactus (IκB homolog) and activation of the transcription factors Dorsal and Dorsal-related Immunity Factor (DIF) (homologs of NF-κB), in development and immunity, respectively.

The N-terminal extracellular domain (ECD) of Toll and TLRs is responsible for the ligand binding. Signaling in turn is achieved via ligand-induced dimerization followed by receptor clustering with cell-specific signaling molecules. The versatile leucine-rich repeat (LRR) motifs in the ECD provide key sites to fulfill these functions. The LRR consensus is typically a short sequence that consists of about 24 residues with leucine residues at conserved positions. Each repeat contributes one turn to the coil that spans throughout the ECD. The conserved leucines participate in the hydrophobic core, whereas the nonconserved residues are surface-exposed and likely candidates for molecular interactions. Toll possesses 21 predicted LRR sequences segregated in two blocks capped by cysteine-rich regions (Figure S1 available online). There are two types of cysteine-rich regions at the N- (LRRNT) and the C terminus (LRRCT) of each block. In Toll, the N-terminal block is involved in ligand binding, whereas the C-terminal one mediates receptor dimerization (Schneider et al., 1991; Weber et al., 2005).

Toll stands out compared to other members of the family with regards to its dual role in development and immunity, its endogenous ligand, and also its heterogeneous stoichiometry (Gangloff et al., 2008; Weber et al., 2005). As for a growing number of cytokine receptors, Toll has been found to form low-affinity dimers with a dissociation constant of 2 μM in the absence of ligand. In the presence of Spz C-106, it is predominantly found in 2:2 complexes with a fraction of 1:1 receptor-ligand associations and trace amounts of 2:1 complexes. This heterogeneity has been attributed to the negative cooperativity of the system (Weber et al., 2005). Remarkably, the closest structural homolog of C-106, NGF has also been described in different ratios with its receptor p75^NTR^ (Aurikko et al., 2005; He and Garcia, 2004).

Previously, we have solved the low-resolution structures of Toll monomers and dimers in the presence and absence of Spz C-106, respectively (Gangloff et al., 2008). In this study we have engaged in truncating the receptor following the hybrid LRR technology (Kim et al., 2007). This strategy proved successful, and crystals that diffracted to 2.4 Å resolution were obtained with a 201-residue-long N-terminal fragment. The crystal structure of the hybrid between Toll (residues 28–228) and variable lymphocyte receptor (VLR) B.61 (residues 133–201) and its functional and biochemical characterization are presented here. To our knowledge, the N-terminal cap adopts a novel fold that is unique to the subtype of Toll-1 receptors in insects. We show that this region is not sufficient for Spz binding in contrast to larger fragments.

## Results

### Determination of the Molecular Structure of the N-Terminal Domain of Toll

The full-length ECD failed to produce material suitable for X-ray crystallography and so did the C-terminal deletion previously characterized, Toll5B (Schneider et al., 1991; Weber et al., 2005). A truncation encompassing the N-terminal domain up to residue Leu 228 was generated according to the hybrid LRR technology and referred to as Toll_N6_-VLR (Kim et al., 2007). This construct was chosen based on the human TLR4 one that yielded structures in the presence and absence of Eritoran-bound myeloid differentiation factor-2 (MD-2) (Kim et al., 2007). The recombinant protein displayed an additional 4 kDa compared to its predicted molecular weight (31 kDa), suggesting that it was modified with carbohydrates. The protein crystallized in the orthorhombic space group P2_1_2_1_2_1_. Molecular replacement with a variety of models and ensembles based on structures of TLRs, glycoprotein Ib α, the Nogo receptor, and the variable lymphocyte receptor (VLR) failed to provide a solution. Experimental phases arose from single-wavelength anomalous dispersion using 5-amino-2,4,6-triiodoisophthalic acid (I3C) (Beck et al., 2008). The native structure was determined to 2.4 Å by molecular replacement using the partial model and refined to an R_work_ of 20.1% (R_free_ of 21.6%) (Table 1). The I3C-bound structure refined at 3.0 Å is described in more detail elsewhere (M.G., A. Moreno, and N.J.G., unpublished data).

### Overall Architecture and Crystal Packing

**Figure 1:**
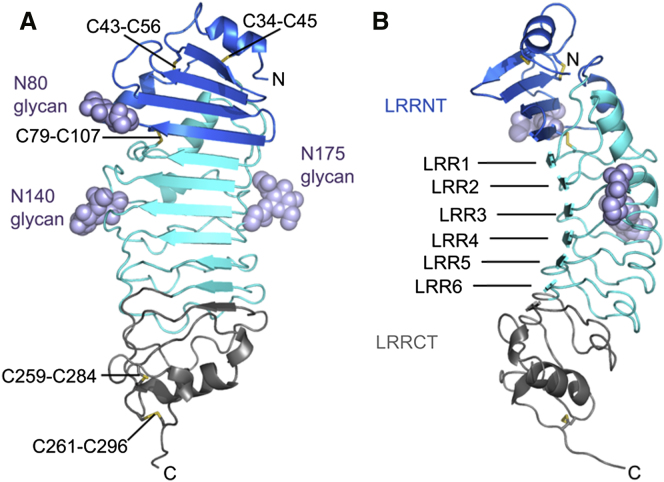
Overall Topology of the Toll_N6_-VLR Hybrid (A) View facing the concave surface of the leucine-rich fold. Glycans attached to asparagine residues 80, 140, and 175 are depicted with light-blue spheres. The Toll LRRNT cap is represented in marine, LRRs are in cyan, and the VLR LRRCT cap is in gray. (B) Left side view showing the curvature of the LRRs. Disulfide bonds are shown as yellow sticks. See also Figure S1.

The crystal structure of Toll_N6_-VLR adopts an arc-shape typical of the leucine-rich repeat (LRR) fold (Figure 1). It is made of an N-terminal capping structure (Ser 28–Leu 94) also known as LRRNT and contains three cystines, followed by five LRRs originating entirely from the Toll receptor and a hybrid one (LRR6) with the junction between Toll and VLR at Leu 228, which is the last Leu in the LRR consensus sequence LxxLxL/xxN, where L stands for Leucine, N for Asparagine, and x for any amino acids (Figure S1). The sixth LRR is tagged along by a cysteine-rich C-terminal capping structure (LRRCT) of VLR B.61. This additional structure is designed to protect the hydrophobic core of the last LRR from solvent exposure. In addition, the structure of this region has helped in other studies to solve the phase problem by molecular replacement with one copy of the molecule in the smallest repeating building block of the crystal, the asymmetric unit (Kim et al., 2007; Yoon et al., 2012).

**Figure 2:**
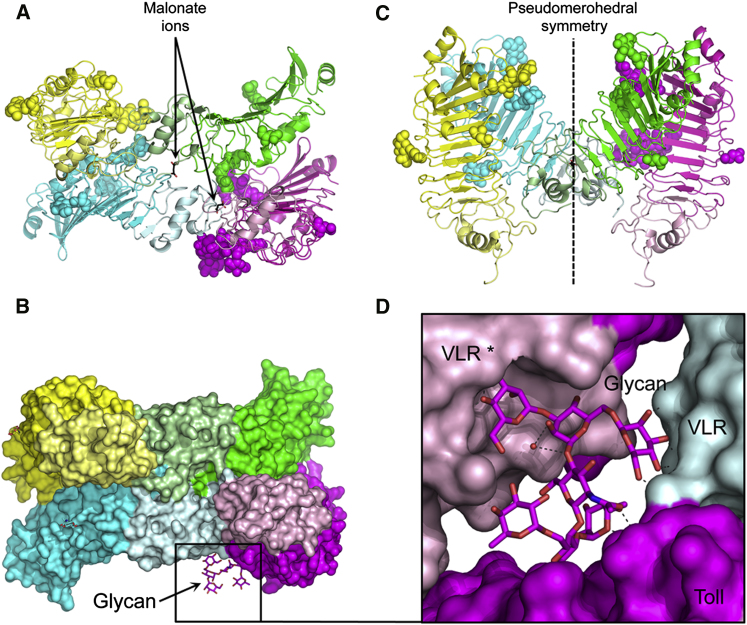
Crystal Packing (A) Asymmetric unit with four molecules of native Toll_N6_-VLR and two malonate ions (MLI). Chain A in green, B in cyan, C in magenta, and D in yellow with the VLR portion in a paler color. (B) View tilted by 90° showing the pseudo 2-fold axis, by which the A-C and B-D pairs are related to each other. (C) Molecular surfaces in the asymmetric unit. The VLR caps mediate most of the crystal contacts. A large glycan structure protrudes from one of the chains and is depicted in magenta sticks. (D) Close-up view on the complex glycan structure bound to Asn 140. See also Table S1 and Figure S3.

In contrast, the asymmetric unit of native Toll_N6_-VLR crystals contains four molecules (Figure 2). Molecules labeled A-C and B-D form pairs that relate to each other by a pseudo 2-fold axis and numerous VLR-mediated contacts. They are head-to-head “spooning pairs” tilted by 40° compared to each other. The tetrameric stoichiometry was detected in solution by dynamic light scattering (DLS) measurements, giving a diameter of 12.7 nm (Z-average size using the cumulant method) and a polydispersion index of about 0.1. In the crystalline form, the size of the four molecules is approximately 45 × 80 × 100 Å^3^, in contrast to the sizes of protomers 25 × 30 × 80 Å^3^ and “spooning” pairs 40 × 55 × 80 Å^3^. The superposition of the dimers displays a root-mean-square deviation (rmsd) of 0.75 Å over 3,942 atoms. The rmsds of superposed protomers are around 0.5 Å between the different molecules. The highest structural divergence is found in the N-terminal loops, in particular at residues Ser 38–Cys 43 and Pro 57–Pro 63.

### Structural Features of the Extended N-Terminal Cap of Toll

**Figure 3:**
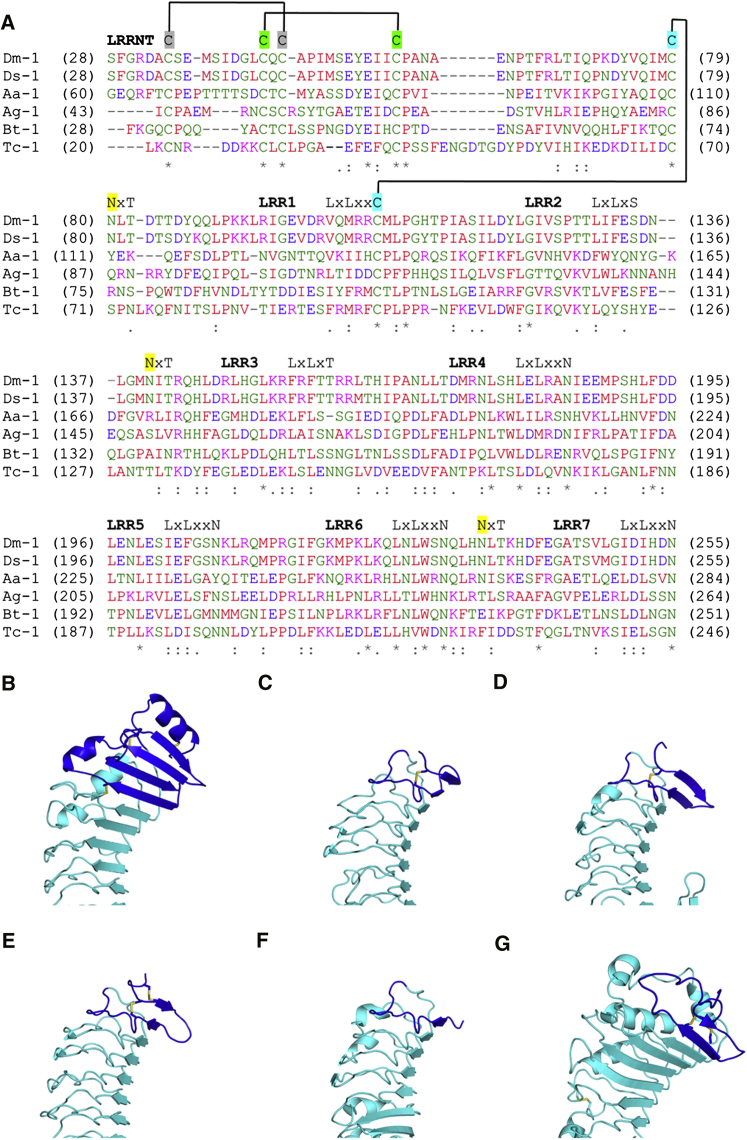
The N-Terminal Cap of Toll Adopts a Unique Fold (A) Sequence alignment of the N-terminal domain of Toll-1 receptor paralogues in insects. *Drosophila melanogaster* Toll (Dm-1); *Drosophila sechellia* (Ds-1); *Aedes aegypti* (Aa-1); *Anopheles gambiae* (Ag-1); *Bombus terrestris* (Bt-1); and *Tribolium castaneum* (Tc-1). (B–G) N-terminal domains of extracellular LRR proteins. (B) Toll, (C) TLR4, (D) glycoprotein Ib α, (E) Nogo receptor, (F) TLR1, and (G) CD14. LRR proteins are shown in the same orientation to highlight the structural diversity of their LRRNTs (in blue). LRRs are represented in cyan, and disulfide bonds are in yellow. See also Figure S2.

Searches using DALI (Holm and Rosenström, 2010) confirmed that the Toll LRRNT adopts a new fold that is shared by Toll-1 paralogues in insects (Figure 3). Toll-like receptors (TLRs) in mammals and Toll orthologs in *D. melanogaster* do not possess this feature (Figure S2). In the crystal structure the N-terminal amphipathic alpha helix (Arg31–Asp40) that is solvent exposed consists of mostly negatively charged residues (Asp32, Glu36, Asp40) and is tethered to the first beta-strand by a disulfide bond (Cys34-Cys45). The extremities of the helix are not well resolved in electron density and have been refined with missing side chains and/or high B factors that reflect the flexibility of this region. Next, the cap forms a four-stranded antiparallel beta-sheet linked by two short turns (two-residue turns) and a seven-residue long loop. This loop separating the duplicated hairpin structures, is located between the second and the third strand of the cap, and adopts different conformations in the different molecules of the a.u. Two additional disulfide bonds stabilize the LRRNT cap in Toll. The covalent bond between Cys43-Cys56 pins down the two first strands, and this structural organization is shared by the LRRNT caps of other extracytoplasmic LRR proteins, such as TLR4 (Kim et al., 2007; Park et al., 2009), glycoprotein Ib α (Huizinga et al., 2002; Uff et al., 2002), Nogo receptor (Barton et al., 2003; He et al., 2003), CD14 (Kim et al., 2005), but is not found in the shorter TLR1 LRRNT (Jin et al., 2007), for instance (Figure 3). The second disulfide bridge between Cys79-Cys107 anchors the fourth strand of the cap to the first leucine-rich repeat (LRR1). This weak bond undergoes radiation damage as suggested by the |F_o_|−|F_c_| electron density.

### Conformational Diversity of the N-Terminal Toll LRRs

**Figure 4:**
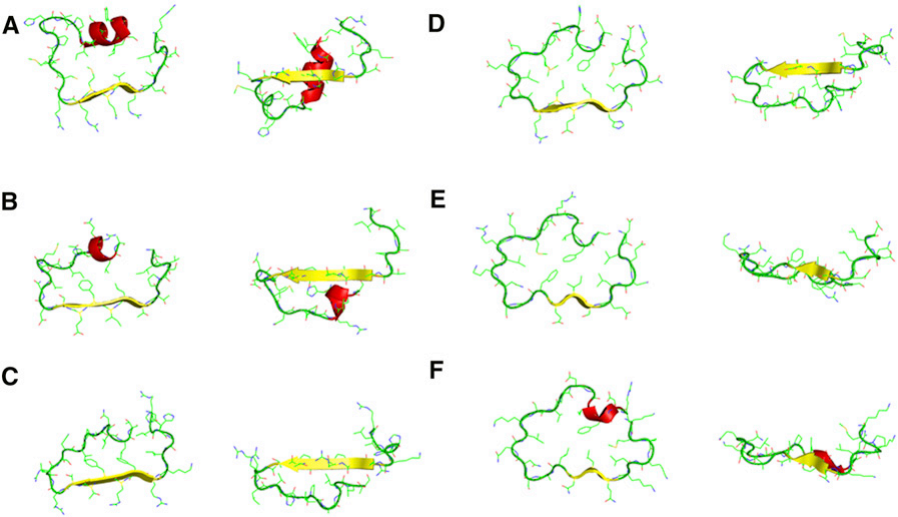
Conformational Diversity of the N-Terminal Leucine-Rich Repeats of Toll (A–F) Each Toll LRR is represented as a cross-section in a planar and a side view at 90°. (F) The hybrid construction in LRR6 is flat, allowing the complete burial of hydrophobic residues in the contiguous LRRs.

Toll LRRs appear irregular in length (between 28 in LRR1 and 23 residues in LRR4) and in secondary structure content (presence of alpha-helices on the convex side in LRR1 and LRR2). The region spanning residues 92 to 359 was nevertheless annotated as a LRR region, but LRR4 was recognized as the first repeat in UniProt (Toll accession code P08953). A parallel beta-sheet defines the concave side and is formed by five-residue-long beta-strands in LRR1–4 and three-residue-long in LRR5 and LRR6. An asparagine ladder usually found in position 13 in the consensus sequence is replaced by Cys, Ser, Thr, or Ala throughout LRR1–4, respectively (Figure S1). Furthermore, a conserved phenylalanine residue features in LRR4, LRR5 and is predicted to occur throughout ten further repeats in the first block of LRRs in Toll to form a hydrophobic spine (Figure S2). The ribbon traces of the repeats observed in the crystal structure go from eight-shaped to flat (Figure 4). The lack of planarity in the first repeats explains why shorter truncations did not express (data not shown). Such fusions would expose hydrophobic patches at the junction between Toll and the flat LRRCT from the VLR even though the prediction of the repeat was correct in terms of primary sequence. This is a perfect illustration of the structural diversity of LRRs. They are easily recognizable in sequence but difficult to predict in three dimensions.

### Glycans Participate in the Crystal Packing

The Toll-VLR construct contains three glycosylation sites located on Toll at Asn 80, 140, and 175 as predicted by the consensus Asn-X-Ser or -Thr sequon with X (any amino acid other than a Pro) (Table S1). Although the first-two glycosylation sites are located on the right flank of the molecule, that is, Asn 80 lies at the end of the fourth strand of the extended LRRNT cap and Asn 140 occurs in LRR2, the Asn 175 residue is found on the left flank in LRR4. Interestingly, the flanks of Toll were overall free of crystal contacts, because of the presence of sugars. The innermost N-acetyl glucosamine (NAG) attached to the Asn residue by a beta (1-4) link was more often observed than subsequent residues, probably because of its lesser degree of flexibility. Carbohydrates could not be added to Asn 140 and Asn 175 in some molecules because of the lack of clear electron density. Mass spectrometry analysis of the Asn 175 confirmed the presence of glycans albeit partially occupied (data not shown). It is, however, not clear if glycosylation promoted crystal packing or contributed to the poor quality of the crystals, as no crystals were obtained with enzymatically deglycosylated protein.

Carbohydrates participate in two types of contacts in the crystal structure; some are involved in protein-sugar interactions and others stack up to form a pattern reminiscent of a “sugar zipper” (Figure 2), a term first quoted by Dorothe Spillmann in reference to cellular recognition (Spillmann, 1994). This occurs on the left flank of each pair and involves the Asn 140 glycan of chains A or D and the Asn 80 of chains C or B. Such interactions are in stark contrast to the crystal packing of the human counterpart TLR4_27-228_-VLR_128-199_ (Kim et al., 2007). This structure was determined in the same space group, as native Toll_N6_-VLR but with only one molecule per asymmetric unit. As a consequence, the packing is much tighter for the latter with a solvent content of about 40% as opposed to 60% for Toll_N6_-VLR. Despite the remote homology and similar hybrid construction, the presence of glycan interdigitation is only observed for Toll-VLR.

Moreover, the glycan structure bound to Asn 140 in chain C is remarkable for two reasons. First, it makes extensive contacts with amino acids of a symmetry-related chain noted C^∗^ at the left flank of the VLR fusion. It is wedged between the two VLR domains, where it forms multiple polar interactions. And, second, an additional fucose residue (Fuc 505 in chain C) is linked by a beta (1-3) bond unique to plants and some insects (Tomiya et al., 2004).

### Protein-Protein Contacts

**Figure 5:**
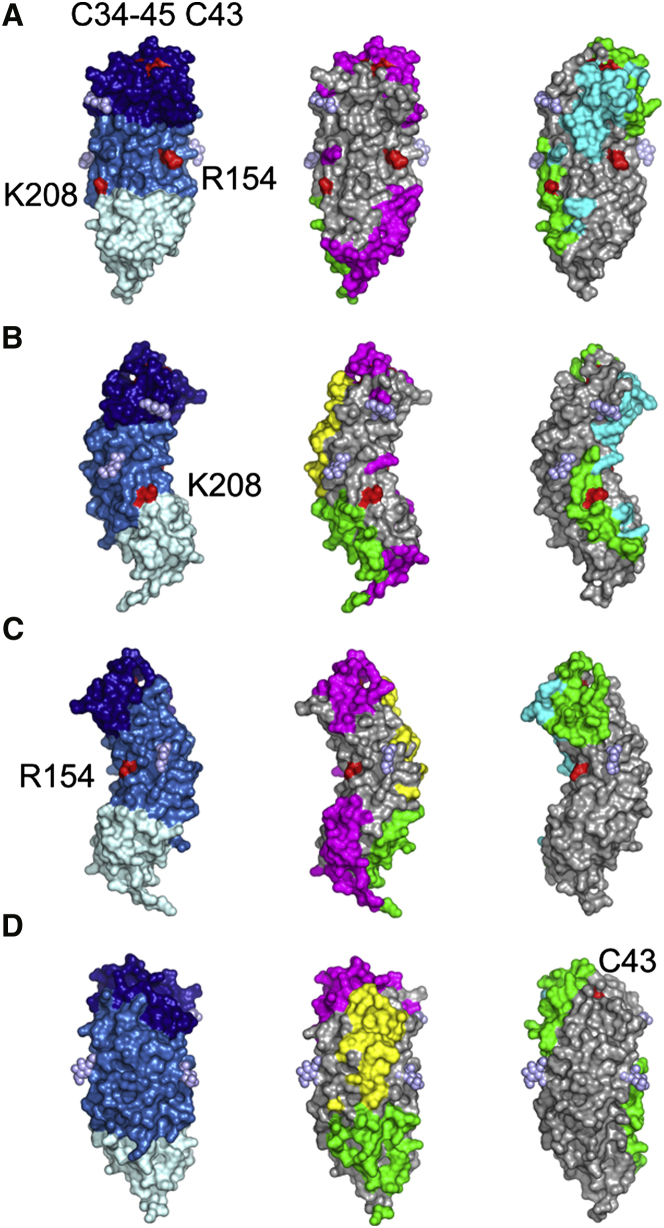
Protein-Protein Contacts (A–D) Areas of crystal contact are indicated on the molecular surfaces of Toll_N6_-VLR in different views. (A) View facing the concave side, (B) right flank, (C) left flank, and (D) convex side. The first column delineates the structural areas in Toll_N6_-VLR with LRRNT in dark blue, LRRs in marine, VLR-LRRCT in light cyan, and glycans in light blue. The second column shows the molecular surface of chain B, and the third is chain D. Contacts are color-coded according to the identity of the molecule that mediates them: chain A is in green, B in cyan, C in magenta, and D in yellow. Residues that have been targeted by site-directed mutagenesis are highlighted in red. See also Figure S3.

To detect functional areas, we decided to more closely inspect crystal contacts mediated by proteins (Figure 5). There are three types of contacts in the asymmetric unit; two of them involve VLR regions. These interfaces have a surface area of approximately 1,110 Å^2^ and 530 Å^2^. Contacts mediated by the VLR region are not of biological relevance to the function of Toll and will not be considered further. The third type of interface occurs between the concave and the convex sides of the A-C and B-D pairs. As it involves the Toll portion of the molecule, it may either reflect the ligand binding or the dimerization mode of the receptor. This interface covers a surface area of 577 and 618 Å^2^ in each pair, respectively. Eight hydrogen bonds stabilize the interface located between the LRRNT and the first 2 LRRs. Such a surface area would be too small to mediate stable ligand binding or receptor dimerization on its own but may be part of a lager network of interactions that have been truncated in the Toll-VLR construct.

In order to determine if such associations are possible in the context of the full-length ectodomain, we overlaid the models of the entire ECD on the truncations. This was achieved by improving our existing ECD model by using the newly characterized N terminus as a template (Gangloff et al., 2008). The Supplemental Experimental Procedures give details of the generation of the model and its structural alignment with the Toll_N6_-VLR crystal structure (Figure S3). We found a number of steric clashes within the A-C and B-D pairs. Glycans that spread throughout the ECD restrict contacts on the flanks and the convex side. The concave side is inaccessible because of glycans on LRRs 7, 11, 16, 18, and 19 (Table S1). More importantly, the translational shift that the ECDs undergo in the concave-convex pause is incompatible with the transmembrane location of the receptor.

In contrast, A and B chains interact at the right flank of LRR6 in a symmetrical way that leaves their C-terminal ends about 220 Å apart. Asymmetric interactions occur between molecules A and D, and also between B and C, at the right flank of LRR2-6 of the latter with LRR7-11 of the former. None of these arrangements is suitable for signaling to the intracellular compartment as the TIR domains are separated by about 200 Å and may illustrate potential quaternary arrangements of Toll in its inactive state.

### Binding Mode of Crystallization Agents

**Figure 6:**
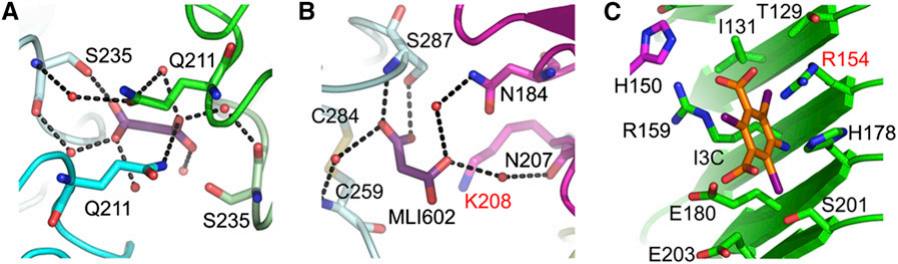
Binding Mode of Crystallization Molecules (A and B) Toll_N6_-VLR binds malonate ions (MLI) in the native structure, and (C) I3C molecules in the derivative. (A) MLI bound at the pseudo 2-fold axis; (B) MLI bound to chain B in blue and C in magenta. (C) I3C binds to the concave side and interacts with residues of the beta-sheet of chain A and the left flank of the chain B. See also Table S2.

The binding mode of small molecules from the crystallization buffer is analyzed to reveal further areas of potential functional importance. Malonate ions (MLI) and I3C were fitted in the electron density of the native and derivative structures (Figure 6). The contacts that these small molecules make with the protein are listed in Table S2. One malonate is located at the pseudo 2-fold axis in a position where it forms hydrogen bonds with Ser 235 and Gln 211 and van der Waals contacts with Val 236 and Pro 237 in both VLR domains of chains A and B. A second malonate nests against the right flank of chains B and C, in the VLR portion of the hybrid molecules, where it makes polar contacts with the hydroxyl of Ser 287 and the carbonyl of its peptide bond, as well as one of the preceding amino acids, Gly 286. It also makes van der Waals contacts with the cystine Cys 259- Cys 284 of VLR LRRCT. Several molecules of water bridge the ion to residues from the Toll portion of the hybrid on the right flank of LRR5 and LRR6. More importantly, the amine moiety of Lys 208 in LRR5 is less than 4 Å away from one of its carboxylates, and the Lys side chain makes van der Waals contacts with it. I3C, the molecule used for phasing, binds at the concave side and interacts with residues from each LRR. It forms a strong hydrogen bond at 3 Å with Arg 154.

### Toll_N6_-VLR Does Not Form a Stable Complex with Spz C-106

**Figure 7:**
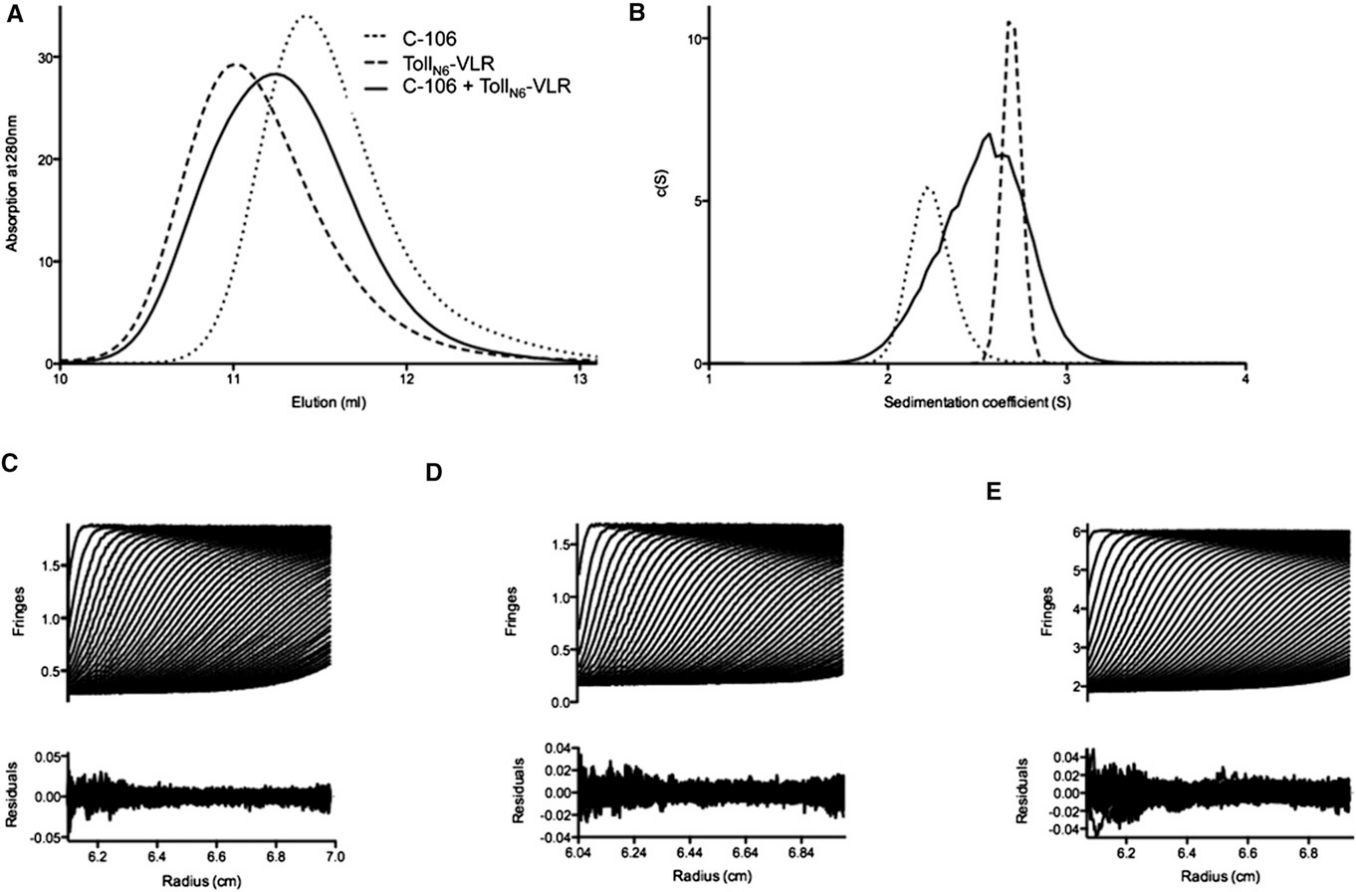
Spätzle Does Not Form a Stable Complex with Toll_N6_-VLR (A) Size-exclusion profiles. Processed Spz C-106 and Toll_N6_-VLR elute in very similar volumes during gel-filtration. (B) Analytical ultracentrifugation profiles. (C–E) Fit and residuals after fitting to a c(s) model in SEDFIT and the distribution of sedimentation coefficients are shown for (C) Spz C-106 at 422 μg.ml−1 (17.6 μM), (D) Toll_N6_-VLR at 377 μg.ml−1 (10.5 μM), and (E) Toll_N6_-VLR and C-106 in equimolar amounts (940 μg.ml−1).

Next, we sought to characterize the complex between Toll and its protein ligand Spz C-106. Whereas Toll_N6_-VLR is an apparent monomer of 40 kDa, upon combining the truncated receptor construct with C-106 in equimolar concentrations, there is no left shift in the chromatogram (Figure 7A). In order to confirm the activity of the ligand, the same preparation of C-106 was used to yield full-length Toll ECD-Spz complexes that elute as 280 kDa molecular species, which corresponds to a 2:2 molecular ratio (data not shown). In the presence of the truncation encompassing Toll residues 28–228, the mixture with C-106 elutes instead in between the elution volumes of the individual proteins. This behavior suggests that either the two proteins do not interact, that they interact with a dramatic change in overall shape, or that they assemble into a transient complex that is not resolved by this technique.

In order to further investigate if C-106 binds Toll_N6_-VLR, we carried out analytical ultracentrifugation (AUC), which can detect changes in shape and stoichiometry in a more sensitive way as these parameters are reflected by differences in the sedimentation coefficient (Figure 7B). The sedimentation velocity method was applied on both proteins in isolation and mixed together in equimolar amounts to determine if a receptor-ligand complex can form in solution. Samples of concentrations between 10.5 μM and 17.6 μM were analyzed, and their sedimentation rates were calculated. The quality of the data is excellent, and the local root-mean-square deviation for each fit was below 0.006. Individually, C-106 and Toll_N6_-VLR have sedimentation coefficients of 2.24 S and 2.69 S, respectively. In contrast to size-exclusion chromatography, AUC detected molecular species with a very broad sedimentation rate peaking at 2.54 S and ranging approximately from 2 S to 3 S. The absence of right shift with the combined proteins suggests that the complex formed with the truncated receptor is unstable.

### Functional Sites Are Located in the N-Terminal LRRs but Not in the Cap

**Figure 8:**
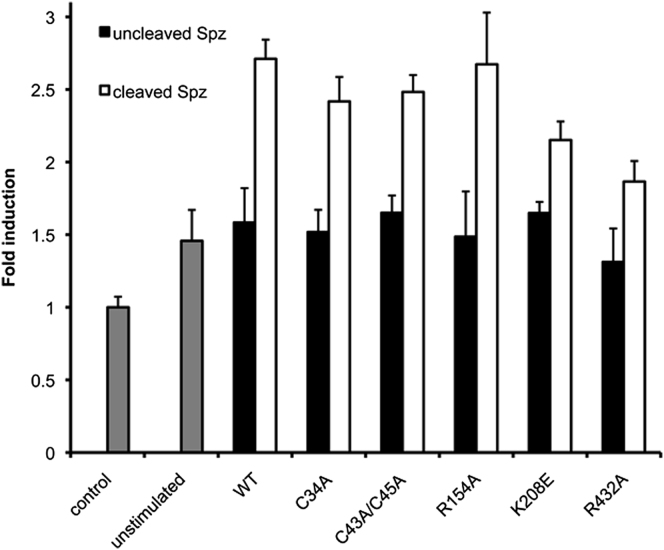
Site-Directed Mutagenesis Confirms that Toll LRRNT Is Not Critical for Signaling HEK293ET cells were transfected with a Toll -TLR4 chimera and a NF-κB luciferase reporter. Luciferase production was measured 24 hr after Spz stimulation at a concentration of 10 nM. Data shown represents fold induction compared with stimulation with media only. Data are represented as means ± SEM. See also Table S3.

Selected residues in the Toll ECD were subjected to site-directed mutagenesis and tested in a cell-based signaling assay to assess their function. The cysteine-rich capping structure has been shown to play a critical role in ligand binding for a number of extracellular LRR receptor, such as mammalian TLRs and the receptor of von Willebrand factor, glycoprotein Ib α (Huizinga et al., 2002). In particular, the TLR4 single mutants, Cys 29 Ala and Cys 40 Ala, and the double mutant Cys 29,40 Ala, were neither coprecipitated with its coreceptor MD-2 nor expressed on the cell surface and failed to transmit LPS signaling (Nishitani et al., 2006). In order to check the role of cysteine residues in Toll, we carried out site-directed mutagenesis on Cys 34, Cys 43, and Cys 45 that form two disulfide bonds Cys 34-Cys 45 and Cys 43-Cys 56. The mutations predicted to be destabilizing (Table S3) were introduced in the Toll-TLR4 chimera that comprises the ECD and the transmembrane region of Toll linked to the TIR domain of human TLR4 (Weber et al., 2005). This construct was found to mediate signaling of an NF-κB reporter gene in response to cleaved Spz in transfected human embryonic kidney HEK293ET cells (Figure 8). The fact that Spz manages to signal in the context of the Toll-TLR4 chimera suggests that the mechanism of signal transduction is conserved in Toll and TLR4. The signal was decreased by about 10% upon introduction of the single and double mutations, which implies that the integrity of the cysteine-rich capping structure affects Toll activation only mildly in contrast to TLR4. Another mutation in the cap involving a negative to a positive charge reversal Glu 36 Arg did not impede signaling either (data not shown).

Next, we chose residues located in crystal contacts and glycan-free areas. We investigated if the buffer binding sites observed in the crystal structure are functionally relevant by carrying out site-directed mutagenesis on Arg 154 located on the convex side of LRR3 and on Lys 208 located on the right flank of LRR5 (Figure 6). The positively charged Arg 154 was substituted by alanine. The mutation was introduced in the Toll-TLR4 chimera and retained wild-type signaling capacities. The charge reversal mutation Lys 208 Glu led to a decrease in signaling by one-fourth compared to the wild-type receptor. This suggests that the malonate-binding site is functionally important. Another site was chosen approximately in the middle of the glycan-bare convex side of Toll ECD. The mutation Arg 432 Ala located on LRR14 decreased the signal by one-third. Interestingly, this residue is located 60 Å away from the previous one and sits across the curved solenoid surface, which increases even further its distance. Spz forms a covalent dimer of approximately 25 × 55 × 60 Å^3^ that cannot reach across both sites unless it does so in the context of a Toll dimer in which it may contact LRR5 on the right flank of one receptor molecule and LRR14 on the convex side of the second one.

### An Antibody that Binds the First Ten LRRs Prevents Signaling

**Figure 9:**
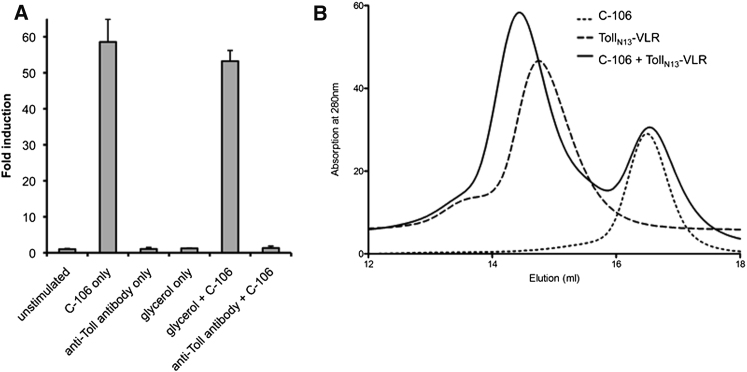
An Antibody that Recognizes the First Ten LRRs of Toll Blocks Spz Signaling (A) The anti-Toll antibody was added to a culture of S2 cells expressing Toll endogenously and which have been stably transformed with a luciferase reporter gene under a drosomycin promoter. Following a 2 hr incubation, 10 nM of cleaved Spz was added to the cells to test for activation and signaling. Controls included glycerol alone, as well as glycerol plus C-106, to ensure that glycerol played no part in the stimulation (when acting as a cryopreservant in the antibody solution), either by activating or inhibiting it. Data is displayed as fold induction compared to media control. Data are represented as means ± SEM. (B) Gel-filtration chromatography revealing that Toll_N13_-VLR is monomeric and binds Spz C106 in a 1:1 complex.

To further investigate the importance of the N-terminal region of Toll, assays were performed using a *Drosophila* S2 cell line that was stably transfected with a luciferase reporter under the control of the *drosomycin* promoter, as previously described (Arnot et al., 2010). Using these cells, maximum activation of the reporter is achieved at 10 nM C-106; thus, this concentration was used for the assay. A 4-fold molar excess of a polyclonal anti-Toll antibody directed against Toll residues 31–330 (Santa Cruz Biotechnology, Santa Cruz, CA, USA) was first added to the cells (this amount was chosen to ensure that the excess of antibody would saturate all binding sites) and following a 2 hr incubation, cleaved Spz was then added to the cells in order to test whether or not signaling would occur (Figure 9A). The antibody completely blocked signaling by either blocking access of Spz to the receptor or by preventing Toll dimerization. As the antibody is directed against the N terminus of Toll up to LRR10, it was important to establish whether this area is able to promote receptor dimerization. This was achieved by characterizing a larger Toll truncation that encompasses the N-terminal region up to Leu 398 in LRR13. This construct is of similar length to the one that was used in the crystal structure of flagellin-bound TLR5 (Yoon et al., 2012). We found that the Toll_N13_-VLR construct remained monomeric in solution and formed a 1:1 complex in the presence Spz C-106 (Figure 9B). Given that the antibody recognizes the receptor in a region unable to mediate dimerization, we conclude that signaling is prevented by competition with ligand binding within the first ten LRRs.

## Discussion

We present the crystal structure of the N-terminal domain of *D. melanogaster* Toll and show that it adopts a fold unique to Toll-1 orthologs in insects. There are nine Toll paralogues in *D. melanogaster* and six Spz isoforms. Functional Toll-Spz pairs other than Toll-1−Spz-1 have not been characterized yet. Toll receptors in *D. melanogaster* belong to three groups based on the architecture of their ECDs (Figure S4). The first group is the largest and includes six receptors 1, 2, 5, 6, and 7, which contain two blocks of LRRs in their ECDs. In contrast, the second group contains Toll-9 that has a single domain of LRRs. Earlier phylogenetic analysis reveal that this receptor and the mammalian TLRs share a common ancestor as they are more closely related to each other than to other insect receptors (Bilak et al., 2003; Gangloff et al., 2003). Finally, the third group of Toll-3 and Toll-4 differs considerably from Toll-1 and TLRs with several shorter LRR domains and no secretion signal. Assuming that Toll-Spz pairs are unique, we speculate that the six Spz isoforms bind the six members of the first group of Toll receptors. None of them share the duplicated LRRNT cap of the prototypical Toll receptor, which our study revealed not to be critically involved in Spz recognition. Additionally, Toll-7 has recently been shown to respond to vesicular stomatitis virus (VSV) glycoprotein G (Nakamoto et al., 2012). In contrast to Toll-1 that exclusively binds Spz-1 (Weber et al., 2003), it is conceivable that Toll-7 might be as promiscuous as mammalian TLR4, which is not only the receptor for LPS but also for VSV (Georgel et al., 2007) and a range of endogenous and exogenous molecules.

As the structural basis for Toll and Spz interaction is still elusive, we used receptor truncations, blocking antibody and site-directed mutagenesis to gain further insight. Schneider and collaborators inferred that Toll truncations encompassing at least the first 463 residues of the Toll ectodomain were sufficient for C-106 binding (Schneider et al., 1991). The ethylmethane sulfonate-induced mutation *Toll84c* (Gln 464 STOP) is a gain-of-function mutant with ventralized phenotype. This effect required the presence of a wild-type Toll protein and upstream genes to exert their ventralizing effect. In the absence of the full-length receptor the truncation produced entirely dorsalized embryos as did knockout *Toll* ^*-*^ embryos. Receptor molecules deleted of their transmembrane and cytoplasmic regions are thought to diffuse the activated Spz ligand to more dorsal positions, where it exchanges with and activates the wild-type receptor. This causes the otherwise spatially restricted Spz signal to be elicited in all parts of the embryo, thus triggering all cells to develop ventral cell fates. Toll residues 28–463 encompassing the first 15 LRRs could not be produced in sufficient amounts for structural characterization. The present study narrows the area required for ligand binding down to the first 13 LRRs with the characterization of a VLR construct containing residues 28–397. An antibody that binds the first ten LRRs blocks Toll signaling, which suggests that access to this area is critical for Spz binding.

Based on the effect of the two mutations located in LRR5 and LRR14 of Toll (Lys 208 Glu and Arg 432 Ala, respectively), we propose a model for Spz C-106 binding in which the ligand promotes receptor binding and crosslinking of a second receptor chain into a ligand-induced receptor dimer, which differs from the dimers found in the absence of ligand. The latter are thought to keep the juxtamembranes sufficiently apart to prevent productive TIR domain dimerization and recruitment of the intracellular signaling machinery. Our revised model suggests that Spz binds across the right flank and convex sides of two Toll ECDs reminiscent of protein-binding TLRs (Park et al., 2009; Yoon et al., 2012). A ligand-independent C-terminal dimerization region is located in the second half of the ECD as suggested by the characterization of a deletion product starting at Asp 458 that forms constitutive dimers (Weber et al., 2005). This establishes crudely the boundary between the N-terminal ligand binding domain and the C-terminal dimerization region at LRR14. Further work is clearly necessary to get a full picture. The determination of the crystal structure of the Toll-Spz complex will be aided by the data presented here.

## Experimental Procedures

### Cell Culture

Sf9 cells were used for baculovirus generation and protein expression. The cells were grown at 28°C in a suspension culture using Sf-900 II SFM (Invitrogen, Carlsbad, CA, USA) supplemented with 0.1% pluronic acid (Sigma-Aldrich, St. Louis, MO, USA). A stable *Drosophila* cell line (648-1B6) expressing luciferase under the control of the drosomycin promoter was established from S2 cells (Invitrogen) and was a kind gift from Jean-Luc Imler (LeMosy et al., 2001). These cells were grown at 28°C in Express Five SFM (Invitrogen), supplemented with 2 mM L-glutamine, 1% penicillin/streptomycin, and 0.5 μM puromycin. HEK293ET (human embryonic kidney 293 EBNA-T) cells were grown at 37°C (5% CO_2_, 100% humidity) in Dulbecco’s modified Eagle’s medium (Invitrogen), supplemented with 10% fetal calf serum (Invitrogen) and 2 mM L-glutamine.

### Site-Directed Mutagenesis

Site-directed mutagenesis was performed using the QuikChange II kit (Stratagene, LaJolla, CA, USA). The primers are listed in Table S1. The mutagenized insert were DNA-sequenced and then recloned into fresh pcDNA3.1(+) and pFastBac-1 backbones as appropriate. Mutations did not affect protein expression levels as assessed by transient transfection of HEK293 cells followed by western blot detection of the receptor and GAPDH as a loading control.

### Luciferase Assay

S2 cells were placed into 96-well plates and stimulated overnight by the addition of purified recombinant Spätzle to the culture medium. Cells were lysed using Passive Lysis buffer (Promega, Madison, WI, USA), and the activity measured using a GloMax luminometer (Promega) immediately after the addition of the D-Luciferin substrate (Biosynth, Itasca, IL, USA). All assays were performed three times in triplicate.

### Generation of Toll Truncations

Toll-VLR constructs encompassing Toll residues 1–228 and 1–397, respectively, were generated as fusions with residues Asn 133–Thr 201 of hagfish VLR B.61 by PCR. Toll truncations carried a 5′-BamHI and a 3′-*NheI* restriction site. The LRRCT of VLR B6.1 was generated with a 5′-*NheI* and a 3′-*AgeI* cloning site. The Fc domain of human IgG1 was amplified with a 5′-*AgeI* site, followed by a TEV cleavage site and 3′-*NotI* site. Primers are listed in Table S4. The Toll, VLR, and Fc fragments were digested with the corresponding restriction enzymes. The three products were then ligated to form a single Toll-VLR-Fc insert that was introduced into the BamHI and *NotI* sites of the pFastBac-1 transposition vector (Bac-to-Bac; Invitrogen).

### Protein Expression and Purification

Fc-tagged Toll N-terminal trunactions and His-tagged Spätzle were produced in a baculovirus expression system. The procedure for Spz preparation has been described elsewhere (Gangloff et al., 2008). Toll-VLR constructs were expressed in Sf9 insect cells (Invitrogen). The supernatant was collected by centrifugation 3 days after infection and concentrated using the Centramate tangential flow filtration system (Pall Filtron, VWR, Radnor, PA, USA). It was loaded onto HiTrap protein A HP column (GE Healthcare, Waukesha, WI, USA). Purified protein was eluted in 0.1 M sodium citrate (pH 3.0). The Fc fusion was cleaved with TEV protease (Kapust et al., 2001). The digestion products were separated by protein A affinity chromatography. Toll-VLR constructs were further purified by size-exclusion chromatography in 100 mM NaCl and 20 mM Tris-HCl (pH 7.0).

### Sedimentation Velocity Analytical Ultracentrifugation

All analytical ultracentrifugation experiments were performed on an Optima XL-A/I (Beckman Coulter, Brea, CA, USA) centrifuge equipped with a four-hole titanium rotor, double-sector centerpieces, and an interference optical system for data acquisition. Sedimentation velocity runs were performed at 45,000 rpm with 3 min intervals between scans for a total of 190 scans at 20°C. The sample volume was 400 μl. Data were analyzed using Sedfit software (Schuck, 2000). The partial specific volumes, buffer density, and viscosity were estimated using SEDNTERP software (Laue et al., 1992).

### Dynamic Light Scattering Characterization

Dynamic light scattering (DLS) was performed using a Zetasizer Nano-S instrument (Malvern Instruments, Malvern, Worcestershire, UK). Protein samples in 100 mM NaCl and 50 mM Tris-HCl (pH 7.0) buffer at an approximate concentration of 200 μg.ml^−1^ were centrifuged for 5 min at 13,000 g to remove any aggregates. Aliquots of 40 μl were then loaded into disposable solvent-resistant micro cuvettes (Malvern), followed by ten DLS measurements, which were averaged to determine the diameter and polydispersion index of the proteins in solution.

### Crystallization of Toll-VLR

Crystals were obtained with the counterdiffusion method in agarose gels (M.G., A. Moreno, and N.J.G., unpublished data). Briefly, 10 μl protein at 23 mg/ml was mixed with 10 μl agarose 0.6%, boiled for 1 min, and cooled to about 40°C before use. A volume of 8 μl protein mixture was introduced in capillary tubes of internal diameter 0.3 mm and length 80 mm (Capillary Tube Supplies, Withiel Bodmin, Cornwall, UK). It solidified within a few minutes, upon which 20 μl sodium malonate 2.4 M (pH 7.0) was added on top of the gel. The capillary was sealed on both ends using plasticine and nail polish. Two weeks later, the precipitant solution was replaced with 10 μl sodium malonate 3.4 M (pH 7.0) and allowed to diffuse for another couple of weeks before harvesting the crystals. The crystal were washed in the already cryoprotecting precipitant solution and mounted in 20 micron nylon cryoloops (Hampton Research, Aliso Viejo, CA, USA) before being immersed in liquid nitrogen for storage and diffraction. Native crystals belong to the space group P2_1_2_1_2_1_ with cell parameters a = 88.79 Å, b = 93.28 Å, c = 225.34 Å, α = β = γ = 90° (four molecules in the asymmetric unit, 62% solvent).

### Heavy-Metal Derivatization

The counterdiffusion method was used for cocrystallization of Toll_N6_-VLR and 5-amino-2,4,6-triiodoisophthalic acid (I3C) in 0.15 M I3C, 2.89 M sodium malonate (pH 7.0) (M.G., A. Moreno, and N.J.G., unpublished data). After an initial diffusion period of two weeks, the precipitant solution was replaced with 20 μl fresh I3C solution and allowed to equilibrate for another couple of weeks. The anomalous signal of the bound iodines was exploited for phase determination using the single anomalous dispersion (SAD) method. Derivative crystals belong to the space group P4_3_2_1_2 with cell parameters a = b = 87.64 Å, c = 220.74 Å, α = β = γ = 90° (two molecules in the asymmetric unit, 59% solvent).

### Data Collection, Phase Determination, and Model Refinement

The structure of Toll_N6_-VLR was determined in two steps. The best SAD data set was collected at a resolution of 3.0 Å at beamline ID23 of the European Synchrotron Radiation Facility (ESRF, Grenoble, France). Oscillation images were integrated, and reflection intensities were merged and scaled using the XDS package (Kabsch, 2010). An electron density examination in Coot (Emsley and Cowtan, 2004) confirmed the hand of the tetragonal space group and allowed manual building of 85% of the molecular model. The partial model was then used for molecular replacement in the 2.4 Å resolution native orthorhombic data set. The final model was obtained after numerous rounds of refinement in Buster (Blanc et al., 2004) and manual rebuilding in Coot. Both 2|F_o_|-|F_c_| and |F_o_|-|F_c_| electron density maps were used in model building. TLS refinement was used with one group per chain. A total of 333 water molecules were built that were within hydrogen bonding distance to the protein. The structure was assessed for correctness and validated using Molprobity (Chen et al., 2010).

## Figures and Tables

**Table 1:** X-Ray Diffraction Data Collection and Refinement Statistics

	Native	Derivative
**Data Collection**		

Space group	P2_1_2_1_2_1_	P4_3_2_1_2
Cell parameters		
a, b, c (Å)	88.79, 93.28, 225.34	87.64, 87.64, 220.74
α, β, γ (°)	90.0, 90.0, 90.0	90.0, 90.0, 90.0
Resolution (Å)	29.9–2.41 (2.54–2.41)^a^	47.40–3.00 (3.16–3.00)^a^
No. observations	481,766	234,278
No. unique reflections	72,966	18,063
R_merge_ (%)^b^	0.056 (0.542)^a^	0.138 (0.653)^a^
I/σ(I)	20.6 (3.0)^a^	
Completeness (%)	99.3 (97.1)^a^	99.9 (100.0)^a^
Mean multiplicity	6.6 (6.0)^a^	13.0 (12.0)^a^

**Refinement**		

Resolution (Å)	29.9–2.41	
No. reflections (total)	72,763	
No. reflections (test)	3668	
R_work_ (%)^c^	20.09	
R_free_ (%)^d^	21.58	
No. atoms	9,283	
Protein	8,715	
Heterogen atoms	245	
Water molecules	323	
Mean B (Å^2^)	66.50	
Rmsds		
Bond lengths (Å)	0.008	
Bond angles (°)	0.97	

a Numbers in parentheses refer to the highest resolution shell.

b R_merge_ = ∑*hkl*(∑*i*(|I *hkl,i*- < I *hkl* > |))/∑*hkl,i* < I *hkl* >, with I *hkl,i* the intensity of an individual measurement of the reflection with Miller indices h, k, and l, and < I *hkl* > the mean intensity of that reflection. Value calculated for I > 3s(I).

c R_work_ = ∑*hkl*(||Fobs*hkl*|−|Fcalc*hkl*||)/|Fobs*hkl*|, where |Fobs*hkl*| and |Fcalc*hkl*| are the observed and calculated structure factor amplitudes.

d R_free_ is calculated as R_work_ with 5% reflections omitted from the refinement process.

## Accession Numbers

The atomic coordinates and structure factors have been deposited in the Protein Data Bank with the accession code 4ARN.

## Supplemental Information

Document S1. Tables S1–S3, Figures S1–S4, and Supplemental Experimental Procedures

Document S2. 3D Molecular Structure of the N-terminal Domain of *Drosophila* Toll, Related to Figure 1 A typical glycosylated monomer is described. It relates to the Results and Discussion sections and, in particular, to Figure 1.
