# Supplementary material for: Functional Insights from the Crystal Structure of the N-Terminal Domain of the Prototypical Toll Receptor
Source: Structure. 2013 Jan 8;21(1):143–53. doi: 10.1016/j.str.2012.11.003 (PMC3542428; doi:10.1016/j.str.2012.11.003)
Supplement: Document S1. Tables S1–S3, Figures S1–S4, and Supplemental Experimental Procedures [file mmc1.pdf]

**Structure, Volume *21***

## Supplemental Information

# Functional Insights from the Crystal Structure of the N-Terminal Domain of the Prototypical Toll Receptor

**Monique Gangloff, Christopher J. Arnot, Miranda Lewis, and Nicholas J. Gay**

**Inventory of Supplemental Information**

***Tables***

**Table S1. Location of glycosylation sites in *D. melanogaster* Toll-1 receptor (relates to Fig. 2 and Fig. S3)**
**Table S2. Binding mode of crystallization agents (relates to Fig. 6)**
**Table S3. Predicted effect of amino acid substitutions on Toll protein stability (relates to Fig. 8)**
**Table S4. Cloning and Mutagenesis primers (relates to the Experimental procedures)**

***Figures***

**Figure S1. *D. melanogaster* Toll-1 LRR alignment (relates to the Introduction and Fig. 1)**
**Figure S2. Sequence alignment of Toll paralogues in *D. melanogaster* (relates to Fig. 3)**
**Figure S3. Structural alignment of full-length Toll ECDs on Toll$_{N6}$-VLR (relates to Fig. 2 and 5)**
**Figure S4. Phylogenetic analysis of Toll paralogues in *D. melanogaster* (relates to the Discussion and Fig. S2)**

***Method***

**Homology modelling of the full-length Toll ectodomain (relates to Fig. 5 and Fig. S3)**

***Supplementary references***

**Table S1. Location of glycosylation sites in *D. melanogaster* Toll-1 receptor.** Glycosylation sites are spread throughout the ECD, except on the convex side, which is extremely smooth up to the juxtamembrane region. Ligand binding contributing to the dimeric interface is foreseen in this area. The left flank contains 4 sites at LRRs 4-7-13-20. Glycans also decorate the concave side where they impede access throughout the ECD due to their location on LRRs 7-11-16-18-19. The 6 first LRRs are free of sugars. This allows protein-protein interactions such as the stacking of the truncations in the crystal structure. The glycosylation pattern of the right flank is interesting as it is very busy, except for an area between LRR6 and 17 free of sugars and potentially accessible for protein-protein interactions.

| Residue | LRR | Left flank | Concave | Right flank | Convex |
|---|---|---|---|---|---|
| 80 | LRRNT | | | X | |
| 140 | 2 | | | X | |
| 175 | 4 | X | | | |
| 235 | 6 | | | X | |
| 270 | 7 | X | | | |
| 275 | 7 | | X | | |
| 346 | 11 | | X | | |
| 391 | 13 | X | | | |
| 482 | 16 | | X | | |
| 508 | 17 | | | X | |
| 528 | 18 | | X | | |
| 654 | LRRNT2 | | | X | |
| 677 | LRR19 | | X | | |
| 703 | LRR20 | | | X | |
| 715 | LRR20 | X | | | |
| 730 | LRR21 | | | X | |
| 738 | LRRCT2 | | | | X |

**Table S2. Binding mode of crystallization agents.** Residues are annotated according to their chain name (A, B, C or D), the 3-letter code of amino acids and to the protein numbering as found in the crystal structures of the native crystal (PDB code, 4ARN) and the I3C-derivative one (PDB code, 4ARR) (Gangloff M., Moreno A., Gay N.J., unpublished data).

| Molecule name | Residue | LRR | Location | H-bond |
|---|---|---|---|---|
| MLI (A601) | A:GLN 211 | 5 | Right flank | - |
| | A:MET 212 | 5 | Right flank | - |
| | A:SER 235 | VLR | | 2.81 |
| | A:VAL 236 | VLR | | - |
| | A:PRO 237 | VLR | | 3.52 |
| | A:ARG 263 | VLR | | - |
| | B:GLN 211 | 5 | Right flank | 3.36 |
| | B:MET 212 | 5 | Right flank | - |
| | B:SER 235 | VLR | | 2.84 |
| | B:VAL 236 | VLR | | - |
| | B:PRO 237 | VLR | | - |
| | B:ARG 263 | VLR | | - |
| MLI (B602) | A:HIS 191 | 4 | Convex | - |
| | B:CYS 259 | VLR | | - |
| | B:SER 260 | VLR | | - |
| | B:CYS 284 | VLR | | - |
| | B:GLY 286 | VLR | | - |
| | B:SER 287 | VLR | | 2.87 |
| | B:ILE 294 | VLR | | - |
| | C:ASN 184 | 4 | Right flank | - |
| | C:LYS 208 | 5 | Right flank | - |
| I3C (A1300) | A:ARG 101 | 1 | Concave | - |
| | A:THR 129 | 2 | Concave | - |
| | A:ILE 131 | 2 | Concave | - |
| | A:ARG 154 | 3 | Concave | 3.09 |
| | A:PHE 155 | 3 | Concave | 3.42 |
| | A:ARG 156 | 3 | Concave | - |
| | A:HIS 178 | 4 | Concave | - |
| | A:LEU 179 | 4 | Concave | - |
| | A:GLU 180 | 4 | Concave | - |
| | A:SER 201 | 5 | Concave | 3.39 |
| | A:GLU 203 | 5 | Concave | - |

**Table S3. Predicted effect of amino acid substitutions on Toll protein stability.** Predicted stability score, analogous to the free energy difference between the wild-type and mutant proteins. Positive value indicates an increase in the thermodynamic protein stability, while negative values indicate a decrease in stability. The stability score was evaluated with the SDM server (http://www-cryst.bioc.cam.ac.uk/~sdm/sdm.php) using the Toll ECD model as input. SDM (Worth et al., 2011) utilizes a statistical approach by fitting variables such as substitution frequencies, distance potentials and residue environments to a potential energy function and this method has been shown to have an accuracy of 74% in predicting the sign of stability change and a linear correlation coefficient of 0.60 between predicted and observed ΔΔG values. A cut-off of 2.0 $kcal.mol^{-1}$ in *pseudo* ΔΔG value indicates a significant effect on protein stability.

The cysteine substitutions were the only ones likely to result in a significantly misfolded protein. Cysteine residues at position 34, 43 and 45 are buried and mutations into Ala were expected to lead to functional changes by preventing disulfide bond formation and causing structural destabilization of the N-terminal cap. However, such a phenotype was not observed. Because cysteine mutant proteins are as active as wild type receptor we infer that their expression, folding, localisation, ligand binding and receptor dimerisation is similar to wild type.

The other mutants at positions 36, 154, 208 and 432 are accessible and predicted not to disrupt the structure. Indeed these mutants were designed using the LRR ECD model as a guide and care was taken to choose surface-exposed sites. Interestingly the substitutions predicted to have stabilizing effects were the ones that showed the strongest phenotypes (Fig. 8). These mutants showed a partial loss of activation that suggests that the effect is restricted to side-chain substitutions without affecting structural integrity.

| Position | Secondary structure | Solvent accessibility | Pseudo ΔΔG ($kcal.mol^{-1}$) | Predicted effect of the mutation |
|---|---|---|---|---|
| C34A | Alpha helix | 2.3 % (buried) | -1.26 | destabilizing |
| E36R | Alpha helix | 82.8 % (accessible) | -0.87 | slightly destabilizing |
| C43A | Bend | 2.6 % (buried) | -1.12 | destabilizing |
| C45A | extended strand | 0.3 % (buried) | -1.26 | slightly destabilizing |
| R154A | extended strand | 35.7 % (partially accessible) | -0.97 | slightly destabilizing |
| K208E | loop or irregular | 23.9% (partially accessible) | 0.75 | slightly stabilizing |
| R432A | alpha helix | 61.7% (accessible) | 0.79 | slightly stabilizing |

## Table S4. Cloning and Mutagenesis primers.

| | Sequence (5'-3') | Orientation | $T_m$ (°C) |
|---|---|---|---|
| Cloning | | | |
| Toll(BamHI-Met1) | ACT ACG GAT CCA TGA GTC GAC TAA AGG GCT CC | Forward | 78 |
| Toll(Leu228-NheI) | TAG CTA GCT AGC AAA TTG AGC TGC TTC AGC TT | Reverse | 74 |
| Toll(Leu397-NheI) | TAG CTA GCT AGC CAG ACG CAG ATC CGT CAG | Reverse | 76 |
| VLR(NheI-Asn133) | ATA GCT AGC AAC CAG CTG AAG TCT GTT CCT GAT GGG ATT TTT GAT CGC CTG ACC AGC TTG CAG AAA ATT TGG CTT CAT ACA | Forward | 91 |
| VLR(Thr200-AgeI) | TTC ACC GGT AGT AGG GCA GAT GAT ACT TCG GAC GGG CTT GCC GGA CCC GGA ACA TTT AGC ACT GCC | Reverse | 94 |
| Fc (AgeI-TEV-E45)* | ACT ACC GGT GAA AAC CTG TAT TTT CAG GGC GAG CCCC AAA TCT TGT GAC AAA | Forward | 85 |
| Fc(K276-XbaI-His×6-STOP-NotI) | ATC CAG CGG CCG CCT AGT GAT GGT GAT GGT GAT GGT G | Reverse | 88 |
| Mutagenesis | | | |
| C43A/C45A | GAG ATG AGC ATA GAC GGA CTG GCT CAG GCT GCA CCC ATA ATG TCG GAA TAC | Forward | 79 |
| E36R | CGG TCG GGA TGC GTG CAG TCG GAT GAG CAT AGA CGG ACT GTG | Forward | 84 |
| C34A | CAT CCT TCG GTC GGG ATG CGG CCA GTG AGA TGA GCA TAG ACG | Forward | 83 |
| R154A | CCG TTT GCA CGG TCT AAA GGC ATT CCG GTT TAC CAC TCG CCG | Forward | 82 |
| K208E | GTC AAT TGA ATT CGG TAG CAA TGA GCT GAG GCA AAT GCC GCG TGG | Forward | 82 |
| R432A | ACT GCG TAC CAT CGA TTC CGC AGC CTT TGT AAG CAC AAA TGG ACT G | Forward | 81 |

* Synthetic Fc construct with Genbank accession code ABI97155.1

**Figure S1. *D. melanogaster* Toll-1 LRR alignment**. The consensus sequence is given with x for any amino acid, L for leucine, N for asparagine and F for phenylalanine. The asparagine ladder is highlighted in cyan and the phenylalanine spine in yellow.

```
LRR
Consensus  xLxxLxxLxLxxNxLxxLPxxLFx
3D         Left-Concave-Right-Convex


>Toll
LRRNT1  28 SFGRDACSEMSIDGLCQCAPIMSEYEIICPANAENPTFRLTIQPKDYVQIMCNLTDTTDYQQLPKKL
LRR1    95 RIGEVDRVQMRRCMLPGHTPIASILDYL
LRR2   123 GIVSPTTLIFE-SDNLGMNITRQHLD
LRR3   148 RLHGLKRFRFT-TRRLTHIPANLLT
LRR4   172 DMRNLSHLELR-ANIEEMPSHLFD
LRR5   195 DLENLESIEFGSNKLRQMPRGIFG
LRR6   219 KMPKLKQLNLWSNQLHNLTKHDFE
LRR7   243 GATSVLGIDIHDNGIEQLPHDVFA
LRR8   277 HLTNVTDINLSANLFRSLPQGLFD
LRR9   291 HNKHLNEVRLMNNRVPLATLPSRLFA
LRR10  317 NQPELQILRLR-AELQSLPGDLFE
LRR11  340 HSTQITNISLGDNLLKTLPATLLE
LRR12  364 HQVNLLSLDLSNNRLTHLPDSLFA
LRR13  388 HTTNLTDLRLEDNLLTGISGDIFS
LRR14  412 NLGNLVTLVMSRNRLRTIDSRAFV
LRR15  436 STNGLRHLHLDHNDIDLQQPLLDIMLQTQINSPFG
LRR16  471 YMHGLLTLNLRNNSIIFVYNDWKN
LRR17  495 TMLQLRELDLSYNNISSLGYEDL
LRR18  518 AFLSQNRLHVNMTHNKIRRIALPEDVHLGEG
LRRCT1 549 YNNNLVHVDLNDNPLVCDCTILWFIQLVRGVHKPQYSRQFKLRTDRLVCSQPNVLEGTPVRQIEPQTLIC
          PLDFSDDPRERK
LRRNT2 631 CPRGCNCHVRTYDKALVINCHSGNLTHVPRLPNLH
LRR1   666 KNMQLMELHLENNTLLRLPSANTP
LRR2   690 GYESVTSLHLAGNNLTSIDVDQLP
LRR3   714 TNLTHLDISWNHLQMLNATVLGFLN
LRRCT2 739 RTMKWRSVKLSGNPWMCDCTAKPLLLFTQDNFERIGDRNEMMCVNAEMPTRMVELSTNDIC
```

**Figure S2. Sequence alignment of Toll paralogues in *D. melanogaster.*** The alignment was generated in Clustal Omega (Sievers et al., 2011). Toll-1 LRRNTs are boxed in yellow, LRRCTs in green. Successive Toll-1 LRRs are highlighted in grey and pink alternatively.

```
Toll        ------------------------------------------------------------
Toll5       ------------------------------------------------------------
18wheeler   ------------------------------------------------------------
Toll8       ------------------------------------------------------------
Toll7       ---------------------------------------LAPKES----ESS--------
Toll6       ------------------------------------TESLSTKHHQQQQLSH--------
Toll9       ------------------------------------------------------------
Toll3       ------------------------------------------------------------
Toll4       MEHSKLWDLRPEVRERRFKWTSDGQQQQQQQLGWCNNKDDPPNSHQKSKSNNDARNLNSR

Toll        ------------SFGRDACSEM------------------------------------SI
Toll5       ------------QIIPLPTFCL------------------------------------GL
18wheeler   ------------------------------------------------------------
Toll8       ---------------------------------------------------LSKTVLYQA
Toll7       ------ASAMLGA-GTGAAATV----SLSGDYSSLLSNVPAASPVPAN-----PSQPSGP
Toll6       ------SNAIMGEAGVSNSQL-------------MQPSTPARTLRPLTAGAGGDPSLYDA
Toll9       ------------------------------AYTE-----------------------FSI
Toll3       ------------------------------------------------------------
Toll4       VRVRARVRVVPGEMRMGDINCSNGLGNIREDYCEIYLD------------------ELGE

Toll        DGLCQCAPIM--------------------------SEYEIICPANAE----------NP
Toll5       SPQCTCAAEG--------------------------NVVRFHCPDEYA----------ML
18wheeler   QQQCNWQYGL--------------------------TTMDIRCSVRAL----------ES
Toll8       PDECRWSGG---G----------------------EHDITLVCHLRTI----------NS
Toll7       ANQCSWSYNG--------------------------T-SSVHCALRLI----------ER
Toll6       PDDCHFMPAAGLD----------------------QPEIALTCNLRTV----------NS
Toll9       QDGLIIE-----------------------------PDSATTSSEEAE----------E-
Toll3       ------------------------------------------------------------
Toll4       NGTCSIANNEVTTEDYQMKLVFLKLEINWTSPVFHGWNIFKICNETDYELVIISVLGIRS

Toll        TFRLTIQPKDYVQIMCNLTDTTDYQQLPKKLRIGEVDRVQMRR--CMLPG---HTPIA--
Toll5       L--EVSEPGA--SLYMSYYASTELQWLPR-FNISSLVKIEFDA--YIFWP---EKFLS--
18wheeler   GT-----------------G-TPLDLQVA-------------------------------
Toll8       EL-----------------ENTNFSVIQP-------------------------------
Toll7       QP-----------------G---LDLQGA-------------------------------
Toll6       EF-----------------DTTNFSVIPA-------------------------------
Toll9       ---VSKERTDLKSLMLKYESDDGNSCLL---------DLIKDE--VIWWQFPNGTLRD--
Toll3       ------------------------------------------------------------
Toll4       EVDMRISPAVQYLSLLGIREISGYDIYLPSVLITEMDVHHANGPKMVTFKYLYDSTVNSV

Toll        ----------SILDYLGIVSPTTLIFE-SDNLGMNITRQHLDRLHGLKRFRFT-----TR
Toll5       ----------DLLKTLGVQTVKTIIFRDRT-LETVVTRDVLNSGNG--------------
18wheeler   ----------EAAGRLDLQCSQELLHASEL------APGLFRQLQKLSELRID-----AC
Toll8       ----------QNTVRLRLECNDALFFQSSL------SPDSFRSLVELRDLTIE-----YC
Toll7       ----------DGSSQLTIQCSELYLFESTL------PVAVFARLQTLEALRLD-----SC
Toll6       ----------EHTIALHILCNDEIMAKSRL------EAQSFAHLVRLQQLSIQ-----YC
Toll9       STKKY-----AHKLYLDLSHG--NLKD---------DSDLFREAKLSRKVTIWRTEVFSA
Toll3       --------------------------------------------SHLRSLTFI-----GF
Toll4       ITNNYIRKTMNNTEKIKIY-----YHNTFEKTTLTMEKNIFHGKNKMSALIFN-----GL

Toll        RLTHIPANLLTDM----RNLSHLEL-----R-ANI----------------EEMP--SHL
Toll5       ------------------------------------------------------------
18wheeler   KLQRVPPNAFEGL----MSLKRLTL-----ESHNAVWGPG---------KTLELH--GQS
Toll8       KLGNLTDGSFRGL----QELRNLTI-----RTHNGDWS-T---------MSLEMA--SNS
Toll7       KLLQLPNNAFEGL----ATLKSLRL-----STHNSEWGPT---------RTLELF--PDS
Toll6       KLGRLGRQVLDGL----EQLRNLTL-----RTHNILWP-A---------LNFEIE--ADA
Toll9       AFNTLTAAPFRTLYSMRESLKLLSL-----RGNNF----------------AELIPDAED
Toll3       QIENLSTKPFAQF----INLKRMVLTNCTVRNLTFLRT--LQKSLEHLELDIDNEVDLKY
Toll4       KIKGLTNNTFENL----TSLNTLIFDNVFLKDLSFLRSSTLQSSLTYCIMKVDNMVDLKS
```

```
Toll        FDDLENLESIEFGSNKLRQMPRGIFGKMPKLKQLNLWSNQLHNLTK--HDFEGATSV---
Toll5       ----------YMETSQPENITTWHFGSVPGLKKFKFFSHVP-ELQE--SIFHGFDTL---
18wheeler   FQGLKELSELHLGDNNIRQLPEGVWCSMPSLQLLNLTQNRIRSAEF--LGFSEKLCAGS-
Toll8       FVEFRQLERLDLSLNNIWLIPDGMVCPLKSLQHLNASYNKIQDISN--FYFSAS------
Toll7       LGGLKQLTDLDLGDNNLRQLPSGFLCPVGNLQVLNLTRNRIRTAEQ--MGFADMNCGA--
Toll6       FSVTRRLERLDLSSNNIWSLPDNIFCTLSELSALNMSENRLQDVNE--LGFRDRSKEPTN
Toll9       FARFVNESRLEASNS----V--PHHCELLLL---HNTTDLYDRECY--LYFNNNTNMGQS
Toll3       FTNFSSLKFMKVRNYIPNKNFTALICTH--------------KNCNFIRGINGLECP---
Toll4       FEKFTNLEIIEVSQYKGFKNFTAFICEPY------------KSHCKFTLGINEVACP---
                                                              :

Toll        ----------------------------LGI-----------------------------
Toll5       ----------------------------RDLHLSV---NVT---TLPGNM-LSTVNGTLK
18wheeler   -----------ALSNANGAVSGGSEL--QTLDVSFN--ELR---SLPDAW-GASRLRRLQ
Toll8       -------------LSSRKARVCGSTL--QSLDLSAN--KMV---SLPTA--MLSALGRLT
Toll7       -----------------GSGSAGSEL--QVLDASHN--ELR---SISESW-GISRLRRLQ
Toll6       GSTESTSTTESAKKSSSSSTSCSLDL--EYLDVSHN--DFV---VLPAN--GFGTLRRLR
Toll9       ITTGRN---------------YTNFI--KVLKDRFDQHGSS---SQSIAWATFPKMPRLV
Toll3       -----------------KLCQCLYIIDDLELNIDCSNLGLLQIPPLPIPIY------GDV
Toll4       -----------------LKCNCSYNRDKSQLEIDCWQKNLTTIPSLPVPKK------GSS
                                          :

Toll        --DIHDNGIEQLPHDVFAHLTNVTDINLSANLFRSLPQGLFDHN-KHLNEVRLMNNRVPL
Toll5       TLTIESPGIVSFGNPLLRELQQLRNLSLA--LIH----PFHERD-K--------------
18wheeler   TLSLQHNNISTLAPNALAGLSSLRVLNISYNHLVSLPSEAFAGN-KELRELHLQGNDL--
Toll8       HLNMAKNSMSFLADRAFEGLLSLRVVDLSANRLTSLPPELFAET-KQLQEIYLRNNSI--
Toll7       HLNLAYNNLSELSGEALAGLASLRIVNLSNNHLETLPEGLFAGS-KELREIHLQQNEL--
Toll6       VLSVNNNGISMIADKALSGLKNLQILNLSSNKIVALPTELFAEQAKIIQEVYLQNNSI--
Toll9       ELDISNCSIEYVSKEAFRNVSNLRRLFMSDNKIMTISHDTFYYV-QGVQYLDLSFTNF--
Toll3       KLNFSNNSLSQLPTMTLPGYKLVKRLDVSRNRLTNLSINHLPA---KLDYLDVSFNEI--
Toll4       ALVFQSNLLAELPDNSLEGYHNLKSLDVSYNQLTSLSVSQLPE---SLHYLDIRHNKI--
               .    :  .    :     :  : ::   :

Toll        ATLPSRLFANQPELQILRLR----------------------------------------
Toll5       -QLQPHFFGSMTNLEEVRLAS---------------------------------------
18wheeler   YELPKGLLHRLEQLLVLDLSGNQLTSHHVD---NSTFAGLIRLIVLNLSNNALTRIGSKT
Toll8       NVLAPGIFGELAELLVLDLASNELNSQWIN---AATFVGLKRLMMLDLSANKISRLEAHI
Toll7       YELPKGLFHRLEQLLVVDLSGNQLTSNHVD---NTTFAGLIRLIVLNLAHNALTRIDYRT
Toll6       SVLNPQLFSNLDQLQALDLSMNQITSTWID---KNTFVGLIRLVLLNLSHNKLTKLEPEI
Toll9       -----LTYSYQLQLPTLEMALSLIYGLKIQ---QNVFKYLPELIYLDLSHSKMTRNSAVA
Toll3       INMGNDVIKYLRTVPIFKQTGNQWTIHCDDKPLLNFFRHLKLII----------RMKSAE
Toll4       TTLSPQVVEYLYSVNVFNQYGNKWSIYCDEYHLQEFFWYKAKLL----------RIKTSK
                         :  .

Toll        ---------------AELQSLPGDL-------FEHSTQ----------------------
Toll5       ---------------AT-SSVNRSM-------FKGTNK----------------------
18wheeler   FKELYF-LQILDMRNNSIGHIEEGA-------FLPLYNLHTLNLAENRLHTLDNRIFNGL
Toll8       FRPLAS-LQILKLEDNYIDQLPGGI-------FADLTNLHTLILSRNRISVIEQRTLQGL
Toll7       FKELYF-LQILNLRNNSIGHIEDNA-------FLPLYNLHTLNLAENRLHTLDDKLFNGL
Toll6       FSDLYT-LQILNLRHNQLENIAADT-------FAPMNNLHTLLLSHNKLKYLDAYALNGL
Toll9       FAHLGDKLKFLSL-----------------------------------------------
Toll3       MKPMFL---------HSLTELPKGFLKFLGKHFIWLGVRKQEYYLINEEQ----------
Toll4       FQTIME---------YIELSSKGSFV---ENF--FVQNIDQLYLEANEDE----------

Toll        --------------------------ITNISLGDNLLKTL---PATLLEHQVNLLSLDLS
Toll5       --------------------------LQ-------------------------LI-KMNG
18wheeler   YVLTKLTLNNNLVSIVESQAFRNCSDLKELDLSSNQLTEV---PEAVQDL-SMLKTLDLG
Toll8       KNLLVLSLDFNRISRMDQRSLVNCSQLQDLHLNDNKLQAV---PEALAHV-QLLKTLDVG
Toll7       YVLSKLTLNNNLISVVEPAVFKNCSDLKELDLSSNQLNEV---PRALQDL-AMLRTLDLG
Toll6       YVLSLLSLDNNALIGVHPDAFRNCSALQDLNLNGNQLKTV---PLALRNM-RHLRTVDLG
Toll9       --------CYTAIPMVSSTIFKNT-VLEGLDLSGNPYLSYNIIDDAFDGIANTLKYLYFE
Toll3       -------------------------LLQSMHRKLNNLNTI----------MSIYKYMEWL
Toll4       -------------------------IIDAFGPSDK---------------YFNLKLMEAL
                                      :
```

```
Toll        NNRLTHL----PDSLFAHTTNLTDLRLEDNLLTGISGDIFSNLGNLVTLVMSRNRLRTID
Toll5       NDDLMEL----PGEIFLDQVNLKTLDLSCNAIVTLHEDVFKGLGNLTLLDLSKNRLTNLS
18wheeler   ENQISEF----KNNTFRNLNQLTGLRLIDNRIGNITVGMFQDLPRLSVLNLAKNRIQSIE
Toll8       ENMISQI----ENTSITQLESLYGLRMTENSLTHIRRGVFDRMSSLQILNLSQNKLKSIE
Toll7       ENQIRTF----DNQSFKNLHQLTGLRLIDNQIGNITVGMFQDLPRLSVLNLAKNRIQSIE
Toll6       ENMITVM----EDSAFKGLGNLYGLRLIGNYLENITMHTFRDLPNLQILNLARNRIAVVE
Toll9       RSNIKDL----E--WSKSLKNLQVLGLAGNNINALTPAMFQSLESLEILDLSSNHVGNWY
Toll3       HRKLIFVNREYDLFYIRQMAAPCPHKCEC---------CY-SRDSLILKIDCRNKFVYNF
Toll4       NHAIWLFSGEFDEIILHHLNSPCPYRCSC---------CF-EWHTGEFLINCRNLSLDIY
            .  :  .                                :           . *

Toll        SRAFVSTNGLRHLHLDHNDIDLQQPLLDIMLQTQINSPFGYMHGLLTLNLRNNSIIFVYN
Toll5       STIFAPLTSLNVLRLNKNSLTAMSPSV---------------------------------
18wheeler   RGAFDKNTEIEAIRLDKNFLT------------DINGIFATLASLLWLNLSENHLVWFDY
Toll8       AGSLQRNSQLQAIRLDGNQLK------------SIAGLFTELPNLVWLNISGNRLEKFDY
Toll7       RGSFDKNFELEAIRLDRNFLA------------DINGVFATLVSLLWLNLSENHLVWFDY
Toll6       PGAFEMTSSIQAVRLDGNELN------------DINGLFSNMPSLLWLNISDNRLESFDY
Toll9       RSAFHNNSALRVLNLRSNTINMLSNE-----------MLKDFERLDY-------------
Toll3       PDIVARN---SR------------------------LMRKQNMSSPMELHLSKNNISNITI
Toll4       PRLPNSIPYKTTLYLDRNEIRKLTNTE--------SLVVAGHASIHKLHMSQNLLRELPL

Toll        D-------WK----------------NTMLQLRELDLSYNNISSLGYED------LAFLS
Toll5       --------------------------------------FQDVVSLNY-------------
18wheeler   AFIPSNLKWLDIHGNYIEALGNYYKLQEEIRVTTLDASHNRITEIGAMSVPNSIELLFIN
Toll8       SHIPIGLQWLDVRANRITQLGNYFEIESELSLSTFDASYNLLTEITASSIPNSVEVLYLN
Toll7       AFIPSNLKWLDIHGNYIEALGNYYKLQEEIRVKTLDASHNRITEIGPMSIPNTIELLFIN
Toll6       GHVPSTLQWLDLHKNRLSSLSNRFGLDSELKLQTLDVSFNQLQRIGPSSIPNSIELLFLN
Toll9       ------------------------------------------------------------
Toll3       AMLPKELRFLDLRFNNLVTLD---------------------------------------
Toll4       HLLPENITYLDVRNNLLKYLD---------------------------------------

Toll        QNRL---------------HVNMTHNKIRRIALPEDVHLGEGYNNNLVHVDLNDNPLVCD
Toll5       ------------------------------------------------------------
18wheeler   NNIIGQIQANTFVDKTRLARVDLYANVLSKISLNALRVAPVSAEKPVPEFYLGGNPFECD
Toll8       DNQISKIQPYTFFKKPNLTRVDLVRNRLTTLEPNALRLSPIAEDREIPEFYIGHNAYECD
Toll7       NNLIGNVQPNAFVDKANLARVDLYANQLSKLQLQQLRVAPVVAPKPLPEFYLGGNPFECD
Toll6       DNLITTVDPDTFMHKTNLTRVDLYANQITTLDIKSLRILPVWEHRALPEFYIGGNPFTCD
Toll9       -------------------------------------------------LSLGDNDFICD
Toll3       ----------------------------------DKVLSYLK--KNSIKTKLSGNPWNCD
Toll4       ----------------------------------DGVIAFLEYRENITKIELSGNPWECN

Toll        CTILWFIQLVRGVHK---------------------------------------------
Toll5       ------IEM---VNT---------------------------------------------
18wheeler   CSMEWLQRINN-LTT---------------------------------------------
Toll8       CNLDWLQKVNR-E-S---------------------------------------------
Toll7       CTMDWLQRINN-LTT---------------------------------------------
Toll6       CNIDWLQKINH-ITS---------------------------------------------
Toll9       CHLRAVVEVAAANNKDADCSYRLLNYSQNAVGEEVISLAESLIIDRKLWQSRYIPWLQRS
Toll3       CKSRSVLSILRDHEP---------------------------------------------
Toll4       CKAKAFLSFLRRHEP---------------------------------------------
                    .

Toll        ----PQYSR---QFKLR-----TDRLVCSQ--PNVLE--------GTPVRQIEPQTLICP
Toll5       ----QFYGA---TLLMN-----YEAVVCTN--DE-----------------------AC-
18wheeler   ----RQHPH---V--VD-----LGNIECLM--PHSRSAP------LRPLASLSASDFVCK
Toll8       ----RTQPQ---L--MD-----LDQIHCRL--AYARGSS------HVSLIEAKSDDFLCK
Toll7       ----RQHPR---V--MD-----MANIECVM--PHARGAA------VRPLSGLRPQDFLCR
Toll6       ----RQYPR---I--MD-----LETIYCKL--LNNRERA------YIPLIEAEPKHFLCT
Toll9       YSNIREFNRANHIIKLRFSSEDYMVAKCSAAQPYHLGDLDGDLTLKFQLLDYEASQYYC-
Toll3       ---------------LE-----YDVTLK--------------------------------
Toll4       ---------------ME-----YETVLR--------------------------------
                           :
```

```
Toll          L-----------DFSDDPRERKCPRGCNCHVRTYD---KALVINCHSGNLT-HVPRLPNL
Toll5         --------------QYKSAEWQCDPRCICWVQRSV---GSLIVDCRGTSLE-ELPDLPRT
18wheeler     YESHCPPTCHCCEYEQCECEVICPGNCSCFHDATW---ATNIVDCGRQDLA-ALPNRIPQ
Toll8         YASHCFALCHCCDFQACDCKMECPDRCSCYHDQSW---TSNVVDCSRASYEQTLPSHIPM
Toll7         YESHCFALCHCCDFDACDCEMTCPSNCTCYHDQIW---STNVVDCGGQQTT-ELPRRVPM
Toll6         YKTHCFAVCHCCEFDACDCEMTCPTNCTCFHDQTW---STNIVECSGAAYS-EMPRRVPM
Toll9         -----FNNTDQLQVD----EL----------------------NC---------------
Toll3         ----------RCNIS----PTDCPDVCVCCLDNLTW--PSFIVDCRGEGLL-QMPSLS--
Toll4         ----------RVEIT----DDKCPEDCICCVDTSNSDSLAYVVDCSGKELS-EIPQLPTP
                                                         :*

Toll          --------------------------------------------HKNMQLMELHLENNTL
Toll5         --------------------------------------------TLLST--VLKVGNNSL
18wheeler     DVSDLYLDGNNMPELEVGHLTGRRNLRALYLNASNLMTLQNGSLAQLVNLRVLHLENNKL
Toll8         DSTQLYLDGNNFRELQSHAFIGRKRLKVLHLNHSRIEVLHNRTFYGLLELEVLQLQSNQL
Toll7         DSSVVYLDGNNFPVLKNHAFIGRKNLRALYVNGSQVAAIQNRTFASLASLQLLHLADNKL
Toll6         DTSELYIDGNNFVELAGHSFLGRKNLAVLYANNSNVAHIYNTTFSGLKRLLILHLEDNHI
Toll9         ------------------------------------------------------------
Toll3         ------------------------------------------------------------
Toll4         TY----------------------------------------------GQTTLVFERNSL

Toll          LRLPS-ANTPGYESVTSLHLAGNNLTSID-VDQLPT-NLTHLDISWNHLQMLNATVLGFL
Toll5         TSLPTVSEHSGYANVSGLFLSDNNLTSLGSGDQLPD-NLTHLDVRGNQIQSLSDEFLLFL
18wheeler     TALEG-TEFRSLGLLRELYLHNNMLTHISNATFEPLVSLEVLRLDNNRLSSLP--HLQY-
Toll8         KALNG-NEFQGLDNLQELYLQHNAIATIDTLTFTHLYHLKILRLDHNAITSFA--VWNFL
Toll7         RTLHG-YEFEQLSALRELYLQNNQLTTIENATLAPLAALELIRIDGNRLVTLP--IWQMH
Toll6         ISLEG-NEFHNLENLRELYLQSNKIASIANGSFQMLRKLEVLRLDGNRLMHFE--VWQLS
Toll9         ------------------------------------------------------------
Toll3         -----------------------------------S-RVTYVDLRNNNLTALSQKNRSSI
Toll4         KKWPS-SLLPGYSSVTRFYLAHNRLSDIDQ---LPD-KLEYLDISNNNFSALDDRVRGFL

Toll          NR--TMKWRSVKLSGNPWMCDCTAKP-LLLFTQDNFERIGDRNEMMCVNAEMPTRMVELS
Toll5         QE--PNNTMTLSLSGNPITCGCESLS-LLFFVRTNPQRVRDIADIVCTKQKKSFQ--QME
18wheeler     ----RHSLQGLTLGRNAWSCRCQQLRELAQFVSDNAMVVRDAHDIYCLDAGIKRELELI-
Toll8         -PSY---LNELRLASNPWTCSCEFIDKLRDYINRHEY-VVDKLKMKCDVISGNSTQQMVI
Toll7         ATHFGTRLKSISLGRNQWSCRCQFLQALTSYVADNALIVQDAQDIYCMAASSGTGSAAL-
Toll6         ANPY---LVEISLADNQWSCECGYLARFRNYLGQSSEKIIDASRVSCIYNNATS----VL
Toll9         ------------------------------------------------------------
Toll3         ----ENRSLKLHLLDNPWSCSCNDIE-KINFMKSVSSSIVDFTEIKCSN-GEKLV--SI-
Toll4         QKRMNSSQLQLSLFGNPWTCRCEDKD-FLVFVKEQAKNIANASAIQCIDTGRSLI--EV-

Toll          TND-IC------------------
Toll5         AFE-LCPSY---------VLLISC
18wheeler     GNL---------------ANGPDC
Toll8         YPGSGEPASL---------PVVQC
Toll7         EDSSSNSGSLEKRELDFNATGAAC
Toll6         REKNGT----------------KC
Toll9         ------------------------
Toll3         NQHIVC------------------
Toll4         EETDIC------------------
```

**Figure S3. Structural alignment of full-length Toll ECDs according to the packing of $Toll_{N6}$-VLR truncations in the asymmetric unit of the native crystal structure.** Models of full-length Toll ECDs were built in Modeller (Supplementary material on page 12). Glycan structures made of two N-acetyl-D-glucosamine residues linked by β 1-4 branching with a fucose bound to the hydroxyl in position α 6 of the first one, and a mannose linked to the second one in β 4, were attached to the asparagines at the glycosylation sites listed in Table S1. Structural alignments of Toll residues 28-228 between $Toll_{N6}$-VLR and Toll full length ECDs were performed in the molecular viewer PyMol by using the align command. **(a)** Side view of full-length Toll ECDs aligned on the $Toll_{N6}$-VLR truncations in the native crystal structure. Chain A in green, B in cyan, C in magenta and D in yellow with the $Toll_{N6}$-VLR portion in darker colours. The residues that have been targeted by mutagenesis are shown in red. **(b)** Top view. **(c)** Chains B and D undergo steric clashes at their C-termini (the distance of 35 Å has therefore been stroke through) and also via the glycans on the right flank of chain D. **(d)** Full length ECD of chain D is shown in the same orientation as in (c). (e) Top view of the symmetric contacts between chains A and B. **(f)** The C-terminal ends of chains A and B are about 220 Å apart. **(g)** Asymmetric contacts between chains A and D with their C-termini separated by about 200 Å.

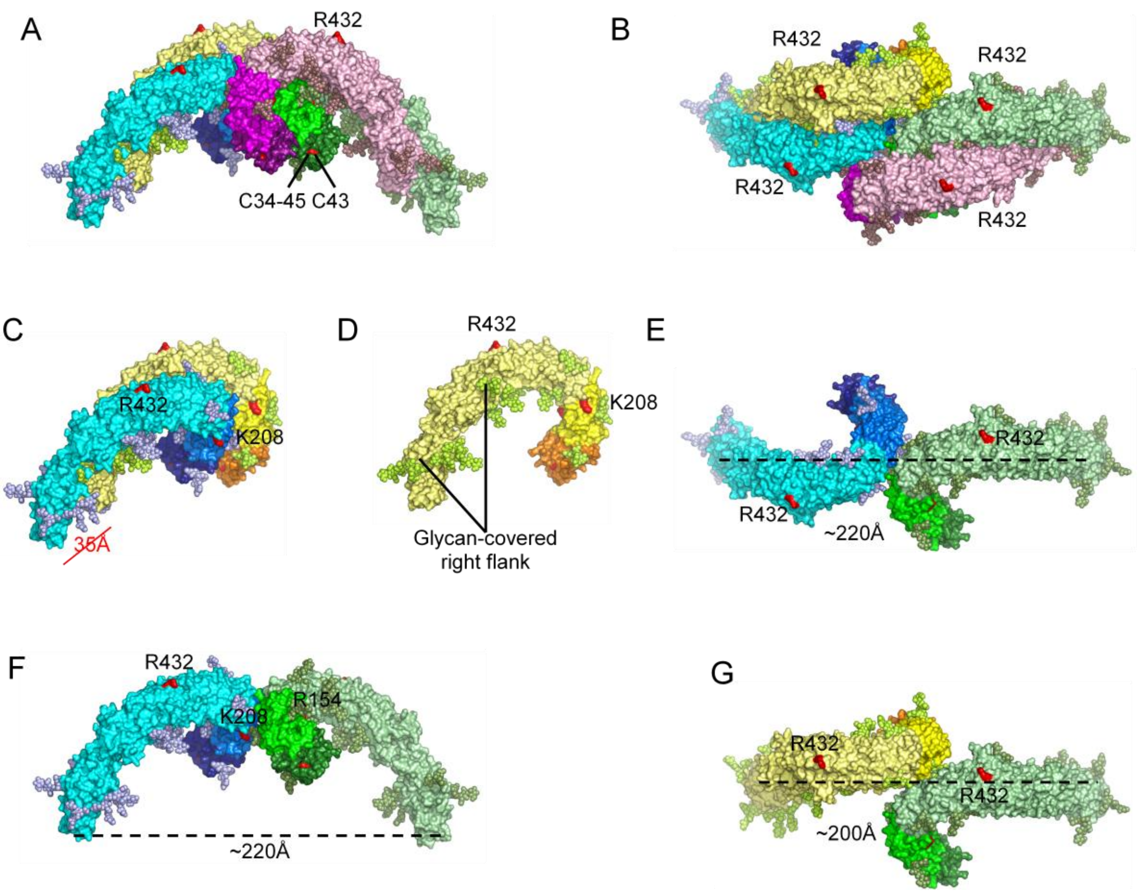

**Figure S4. Phylogenetic analysis of Toll paralogues in *D. melanogaster.***

Cladogram based on the sequence alignment in Fig. S2 showing group I Toll receptors in blue, group II in green and group III in pink. 18Wheeler is also known as Toll2, Toll5 as Tehao and Toll8 as Tollo.

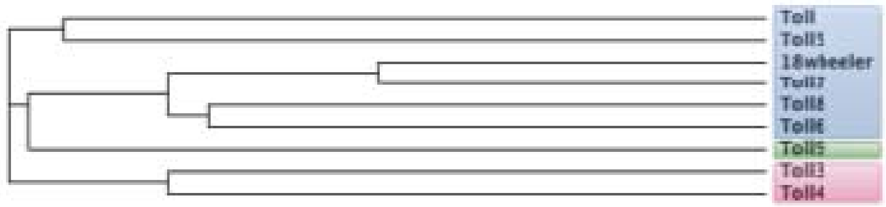

**Supplementary method: Homology modelling of the full-length Toll ectodomain**

Toll sequence was obtained from the Swissprot database with the accession number P08953. The previously generated Toll ECD model based on EM data was modified to incorporate the new structural data generated in this study (Gangloff et al., 2008). Briefly, LRR recognition was aided by the secondary structure prediction programme Psipred (McGuffin et al., 2000) and by inspection of Swissprot sequence annotation. Identification of LRR repeating structural motifs was carried out using FUGUE (Shi et al., 2001), a programme that scans a database of structural profiles, and aligns the query sequence with potential homologues. The Toll ECD was divided into five overlapping areas each modelled separately on the Nogo receptor crystal structure (PDB code 1OZN) except for the N-terminal domain (residues 28-228) taken from the Toll-VLR crystal structure (PDB code 4ARN). Homology models were built using the programme Modeller (Sali and Blundell, 1993). The best models were chosen based on lowest energies and lowest violations as calculated by Modeller. The overlapping areas were then aligned in Coot (Emsley and Cowtan, 2004) and energy minimized in Modeller. Both blocks of LRRs were aligned to form a horseshoe shaped solenoid with a parallel β-sheet on the concave side. The junction between the two LRR blocks devoid of secondary structure elements according to Psipred were modelled *ab initio* with the loop building module of Modeller. Quality assessment of the final model was performed using Verify3D (Eisenberg et al., 1997) and Ramachandran plot analysis was carried out using Molprobity (Chen et al., 2010).

**Supplementary references**

Chen, V.B., Arendall, W.B., 3rd, Headd, J.J., Keedy, D.A., Immormino, R.M., Kapral, G.J., Murray, L.W., Richardson, J.S., and Richardson, D.C. (2010). MolProbity: all-atom structure validation for macromolecular crystallography. Acta Crystallogr D Biol Crystallogr *66*, 12-21.

Eisenberg, D., Luthy, R., and Bowie, J.U. (1997). VERIFY3D: assessment of protein models with three-dimensional profiles. Methods Enzymol *277*, 396-404.

Emsley, P., and Cowtan, K. (2004). Coot: model-building tools for molecular graphics. Acta Crystallogr D Biol Crystallogr *60*, 2126-2132.

Gangloff, M., Murali, A., Xiong, J., Arnot, C.J., Weber, A.N., Sandercock, A.M., Robinson, C.V., Sarisky, R., Holzenburg, A., Kao, C., and Gay, N.J. (2008). Structural insight into the mechanism of activation of the Toll receptor by the dimeric ligand Spatzle. J Biol Chem *283*, 14629-14635.

McGuffin, L.J., Bryson, K., and Jones, D.T. (2000). The PSIPRED protein structure prediction server. Bioinformatics *16*, 404-405.

Sali, A., and Blundell, T.L. (1993). Comparative Protein Modeling by Satisfaction of Spatial Restraints. Journal of Molecular Biology *234*, 779-815.

Shi, J.Y., Blundell, T.L., and Mizuguchi, K. (2001). FUGUE: Sequence-structure homology recognition using environment-specific substitution tables and structure-dependent gap penalties. Journal of Molecular Biology *310*, 243-257.

Sievers, F., Wilm, A., Dineen, D., Gibson, T.J., Karplus, K., Li, W., Lopez, R., McWilliam, H., Remmert, M., Soding, J*., et al.* (2011). Fast, scalable generation of high-quality protein multiple sequence alignments using Clustal Omega. Mol Syst Biol *7*, 539.

Worth, C.L., Preissner, R., and Blundell, T.L. (2011). SDM--a server for predicting effects of mutations on protein stability and malfunction. Nucleic Acids Res *39*, W215-222.
